# Immune Control of Animal Growth in Homeostasis and Nutritional Stress in *Drosophila*

**DOI:** 10.3389/fimmu.2020.01528

**Published:** 2020-07-31

**Authors:** Preethi P, Ajay Tomar, Sukanya Madhwal, Tina Mukherjee

**Affiliations:** ^1^Institute for Stem Cell Science and Regenerative Medicine (inStem), Bangalore, India; ^2^The University of Trans-Disciplinary Health Sciences and Technology, Bangalore, India; ^3^Manipal Academy of Higher Education, Manipal, India

**Keywords:** myeloid cells, high sugar, metabolism, inflammation, stress, innate immunity, insulin

## Abstract

A large body of research implicates the brain and fat body (liver equivalent) as central players in coordinating growth and nutritional homeostasis in multicellular animals. In this regard, an underlying connection between immune cells and growth is also evident, although mechanistic understanding of this cross-talk is scarce. Here, we explore the importance of innate immune cells in animal growth during homeostasis and in conditions of nutrient stress. We report that *Drosophila* larvae lacking blood cells eclose as small adults and show signs of insulin insensitivity. Moreover, when exposed to dietary stress of a high-sucrose diet (HSD), these animals are further growth retarded than normally seen in regular animals raised on HSD. In contrast, larvae carrying increased number of activated macrophage-like plasmatocytes show no defects in adult growth when raised on HSD and grow to sizes almost comparable with that seen with regular diet. These observations imply a central role for immune cell activity in growth control. Mechanistically, our findings reveal a surprising influence of immune cells on balancing fat body inflammation and insulin signaling under conditions of homeostasis and nutrient overload as a means to coordinate systemic metabolism and adult growth. This work integrates both the cellular and humoral arm of the innate immune system in organismal growth homeostasis, the implications of which may be broadly conserved across mammalian systems as well.

## Introduction

The immune system comprises circulating cells and blood-forming tissues whose main function is combating infections. The development of this system is metabolically expensive and often associated with trade-offs with other physiological functions such as reproductive fitness (1, 2) and survival, especially in conditions of nutrition challenge (3). Development of a robust immune system and its impact on animal growth has been described in several studies across animal models. Decreased immune function in flies with increased body mass (4) or improved resistance with reduced competitive ability on a poor diet (5) are some studies illustrating this robust connection. Nevertheless, any understanding of animal growth from the standpoint of immune homeostasis is poorly explored. *Drosophila* is a well-established and a conserved model system for addressing questions pertinent to blood development (6) and mechanisms regulating organismal growth (7) mechanisms. In this study, we have used *Drosophila* to explore the consequences of altering immune homeostasis early in animal life on organismal metabolism and growth control and the implications of nutrient overload in this phenomenon.

*Drosophila* blood cells akin to vertebrate myeloid cells perform functions central to the maintenance of general animal physiology that includes wound healing response (8), antimicrobial functions (9), hypoxia response (10), innate immunity, and response to wasp-parasitization (11). Of the three different types of blood cells prevailing within the *Drosophila* larvae, the platelet-like crystal cells are implicated in wound healing and hypoxia response, whereas lamellocytes are involved in the response to parasitic wasps. The phagocytic plasmatocytes constitute 95% of the differentiated mature cell type. These phagocytic blood cells, akin to vertebrate macrophages, perform functions relevant for clearance of apoptotic cells and invading particles, neuronal pruning, tissue remodeling, and antimicrobial functions (12). Immune cells in *Drosophila* are derived from immune progenitor cells whose development, much like in vertebrates, is derived from two distinct waves of hematopoiesis: the primitive and the definitive. The primitive wave of hematopoiesis occurs in the early embryonic stage where the first pool of blood precursors gets specified from embryonic head mesoderm (13, 14). These hematopoietic precursors proliferate and differentiate into mature hemocytes and constitute the larval circulatory and sessile pools of blood cells detected in the larvae (15) and later in adult stages (12). Definitive hematopoiesis initiates in a larval hematopoietic organ called the lymph gland, which gets specified at the late stages of embryonic development (13, 14). The lymph gland comprises multipotent undifferentiated blood progenitor cells that proliferate and mature to give rise to differentiated blood cells during larval stages of development. By the early pupal stage, the blood progenitor cells completely differentiate, after which the lymph gland disintegrates to release these mature hemocytes into circulation contributing to immune cells in the adult fly (6, 12). By early pupal stage, blood cells complete differentiation into hemocytes, after which the lymph gland disintegrates to release these cells into circulation in the adult fly.

The cues that regulate blood development and homeostasis in *Drosophila* are of both local as well as systemic origin (6). The systemic cues include environmental (odors and sensory stimulation) (16, 17) and of nutritional origins (18), the latter, more relevant to this study. During blood development, blood progenitor cells directly sense amino acids and insulin to sustain their maintenance. Starvation or loss of insulin signaling results in the differentiation of progenitors and activation of inflammatory responses, recapitulating a diabetic-like condition (18). Nutrient-rich conditions (19) or any change in the physiological state of the developing larvae (3) have been shown to alter immune cell numbers as well. As immune cells undergo functional maturation, the macrophage-like plasmatocytes perform lipid-scavenging functions and exert systemic control on glucose homeostasis and survival on lipid-rich diet (20). Taken together, these studies provide evidence of nutrient-dependent modulation of immune cell development, homeostasis, and signaling. What remain unclear are the underlying contributions of the immune cell changes on animal physiology in modulating nutrient conditions. We hypothesize immune cells as effectors of coordinating metabolic homeostasis under these conditions and they are necessary for organismal homeostasis.

Animal growth is a complex adaptive process that is dependent on extrinsic nutrient conditions, and is intricately linked with cues of both developmental (21, 22) and nutritional origins (7, 23, 24). These cues coordinate a central growth program that ensures animals achieve their size and proportion within their respective developmental time scale (21). This cross-talk is facilitated by long-range signaling molecules originating from the brain and fat body in *Drosophila* to coordinate the scaling of animal size in response to nutrient availability (7) uptake, and utilization (21, 25). Indeed, several recent studies have demonstrated a complex interplay of insulin signaling with innate immune pathways in growth and nutritional homeostasis (26–29). These studies have positioned the fat body as the major organ responsible for sensing and storing nutrients, in addition to its immune effector functions as a central member of the innate immune system.

Cells of the innate immune system also sense microbial load, integrate metabolic inputs, and alter nutrient allocation and organismal growth when performing pathogenic clearance functions (3, 30, 31). These functions are very similar to roles performed by the fat body, and we hypothesize immune cells as regulators of metabolism and organismal metabolic homeostasis not only in conditions of immune challenge but also in homeostasis or modulating nutrient environments. Development of metabolic disorders such as diabetes and obesity with altered immune cell activity and function are in agreement with this idea (32). In this study, we test this hypothesis and observe that blood cells are necessary to coordinate systemic metabolism and animal growth in homeostasis and in conditions of nutrient overload. Loss or gain of blood cells early in larval development affected adult growth. Our experiments suggest a role for blood cells in the control of fat body innate immune homeostasis and insulin sensitivity. These findings indicate that immune cell activity, as opposed to their number, orchestrates organismal growth homeostasis especially in conditions of dietary excess.

## Results

### *Drosophila* Larval Blood Cells Function to Control Growth of Adult Flies

**Figure 1:**
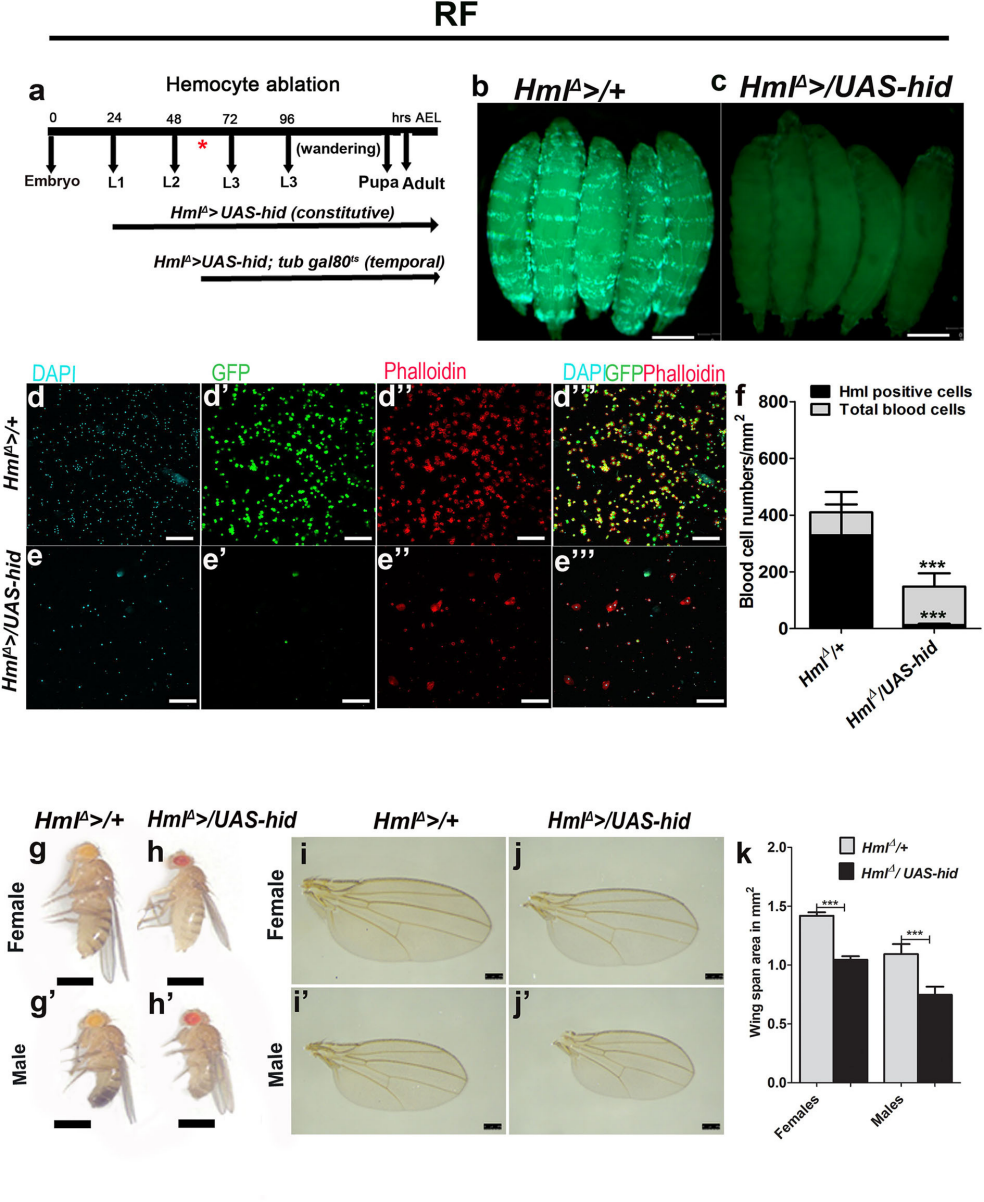
Ablating *Drosophila* larval blood cells leads to adult fly growth defect. In **(b,c,g–h′)**, scale bar = 1 mm, **(d–e′′′)** = 40 μm, and **(i–j′)** = 250 μm. RF indicates regular food. In **(f,k)** bar graphs represent mean ± standard deviation (SD). Statistical analysis applied in **(f,k)** is unpaired *t*-test, two-tailed. “*n*” is the total number of larvae analyzed and “*N*” is the total number of repeats. **(a)** Pictorial representation of the constitutive and temporal experiments undertaken using “*Hml*^Δ^ >” and “*Hml*^Δ^ >*; tub gal80*^*ts*^,” respectively. **(b–f)** Overexpression of “hid” in Hml-positive cells (*Hml*^Δ^ >*UAS-GFP/UAS-*hid) leads to efficient killing of blood cells. **(b)** Control (*Hml*^Δ^ >*UAS-GFP*) larvae depicting blood cells (Hml, green), **(c)** *Hml*^Δ^ >*UAS-GFP/UAS-*hid larvae show lack of GFP expression. **(d–e′′′)** Blood cell characterization in **(d-d′′′)** control and **(e–e′′′)** *Hml*^Δ^ >*UAS-GFP/UAS-hid* larvae reveals reduction in total blood cell numbers, but this does not lead to a complete loss in blood cells as evident from **(d,e)** DAPI, **(d′,e′)** Hml expression (*Hml*^Δ^ >*UAS-GFP*), and **(d″,e″)** Phalloidin stainings. **(d′′′,e′′′)** Merge of all the channels. **(f)** Graphical representation of total and Hml^+^ blood cells in control, *Hml*^Δ^ >*UAS-GFP*, and *Hml*^Δ^ >*UAS-GFP/UAS-hid* larvae. Control (*Hml*^Δ^ >*UAS-GFP/*+) total number of blood cells/mm^2^ (*n* = 16, 409.8 ± 149.4) and Hml-GFP positive cells/mm^2^ (*n* = 16, 328.8 ± 108.8). *Hml*^Δ^ >*UAS-GFP/UAS-hid* show significantly less number of total blood cells/mm^2^ (*n* = 16,148.3 ± 50.04, ***p-value = 0.0003, in comparison with control total blood cells) and less Hml-GFP-positive cells (*n* = 16, 13.11 ± 4.0, ***p-value <0.0001 in comparison with control Hml^+^ cells). **(g–k)** Blood cell ablation affects adult growth. **(g–h′)** Representative images showing adult size defect. In comparison with **(g,g′)** control adult (*Hml*^Δ^ >*/*+) **(g)** female and **(g′)** male fly, **(h, h′)** *Hml*^Δ^ >*/UAS-hid* **(h)** female and **(h′)** male adult flies are smaller in size. **(i–j′)** This growth defect is also shown seen in wing span areas. Representative adult wings from **(i,i′)** controls (*Hml*^Δ^ >*/*+) and **(j,j′)** *Hml*^Δ^ >*/UAS-hid* adults. **(k)** Quantification of wingspan areas. *Hml*^Δ^ >*/*+ (female *n* = 100, 1.42 ± 0.03, male *n* = 1.00, 1.1 ± 0.08) and *Hml*^Δ^ >*/UAS-hid* (female *n* = 100, 1.04 ± 0.03 (***p-value < 0.0001), male *n* = 100, 0.74 ± 0.07 (***p-value < 0.0001).

We aim to explore non-immune homeostatic functions of mature immune cells. To initiate this investigation, the impact of hemocyte ablation from early *Drosophila* larval stages on larval metabolism and development was assessed. *Hemolectin*^Δ^*Gal4 (Hml*^Δ^ >*)* (Figure 1a) was used as the driver to express the pro-apoptotic gene, *hid* in blood cells. Expressing *UAS-hid* specifically in blood cells leads to killing of a majority of blood cells (Figures 1b–f) (33). *UAS-hid* control larvae showed no changes in blood cell numbers or overall larval growth (Supplementary Figures 1a–c), confirming that the dramatic loss of immune cells was specific to *UAS-hid* expression and not a consequence of leaky or non-autonomous *UAS-hid* transgene expression. We ensured the specificity of *Hml*^Δ^*Gal4* driver line by conducting lineage analysis using G-TRACE (34). This approach confirmed *hid* expression (both in real time and lineage based) in blood cells alone, without any expression detected in other larval tissues (Supplementary Figures 1d–h″). *Hml*^Δ^*Gal4* is seen in differentiating and mature populations of larval immune cells, which are essentially the plasmatocytes (Supplementary Figures 1i–i″) (35, 36), making *Hml*^Δ^*Gal4* a reliable driver line to specifically modulate immune cells and assess systemic changes.

Driving *UAS-hid* with *Hml*^Δ^ >resulted in an overall reduction in blood cells. We report a dramatic loss of *Hml* positive (*Hml*^+^) hemocytes (Figures 1b–f) with a small increase in *Hml* negative (*Hml*^−^) blood cells (Figures 1d–f). These results are consistent with published reports (33). Loss of *Drosophila* larval hemocytes using this strategy dramatically affected adult fly sizes (Figures 1g–h′). This was not a consequence of any major defect in larval development in *Hml*^Δ^ >*/UAS-hid* animals (Figure 1c compared with Figure 1b). However, any minor differences cannot be ruled out. To estimate the degree of adult growth retardation, wing span areas of both female and male flies were measured. We observed a significant reduction in wing span areas of *Hml*^Δ^ >*/UAS-hid* adults (Figures 1i–k), indicating a role for *Hml*^+^ blood cells in animal growth control.

Next, we assessed the temporal requirement of larval blood cells in growth control. To address this, we conducted blood cell ablations at mid L2/early L3 time point. Using the temperature-sensitive form of *Gal80*, the expression of *Hml*^Δ^ > was regulated to drive *UAS-hid* transgene expression from mid L2 larval time point until wandering L3 after which these animals were dissected (Supplementary Figure 2a). While this temporal expression of *UAS-hid* was sufficient to successfully eliminate *Hml*^+^ larval blood cells (Supplementary Figures 2a–c), the conditional loss of blood cells post L2 phase of larval development did not result in defective adult growth (Supplementary Figures 2d–g′), as assessed by adult fly sizes (Supplementary Figures 2d–e′) or wing span areas (Supplementary Figures 2f–h). Together, these results show that Hml^+^ cells play a critical role in the systemic control of animal growth at the early phase of the larval development rather than later in larval life.

### Blood Cells Regulate Insulin Signaling

**Figure 2:**
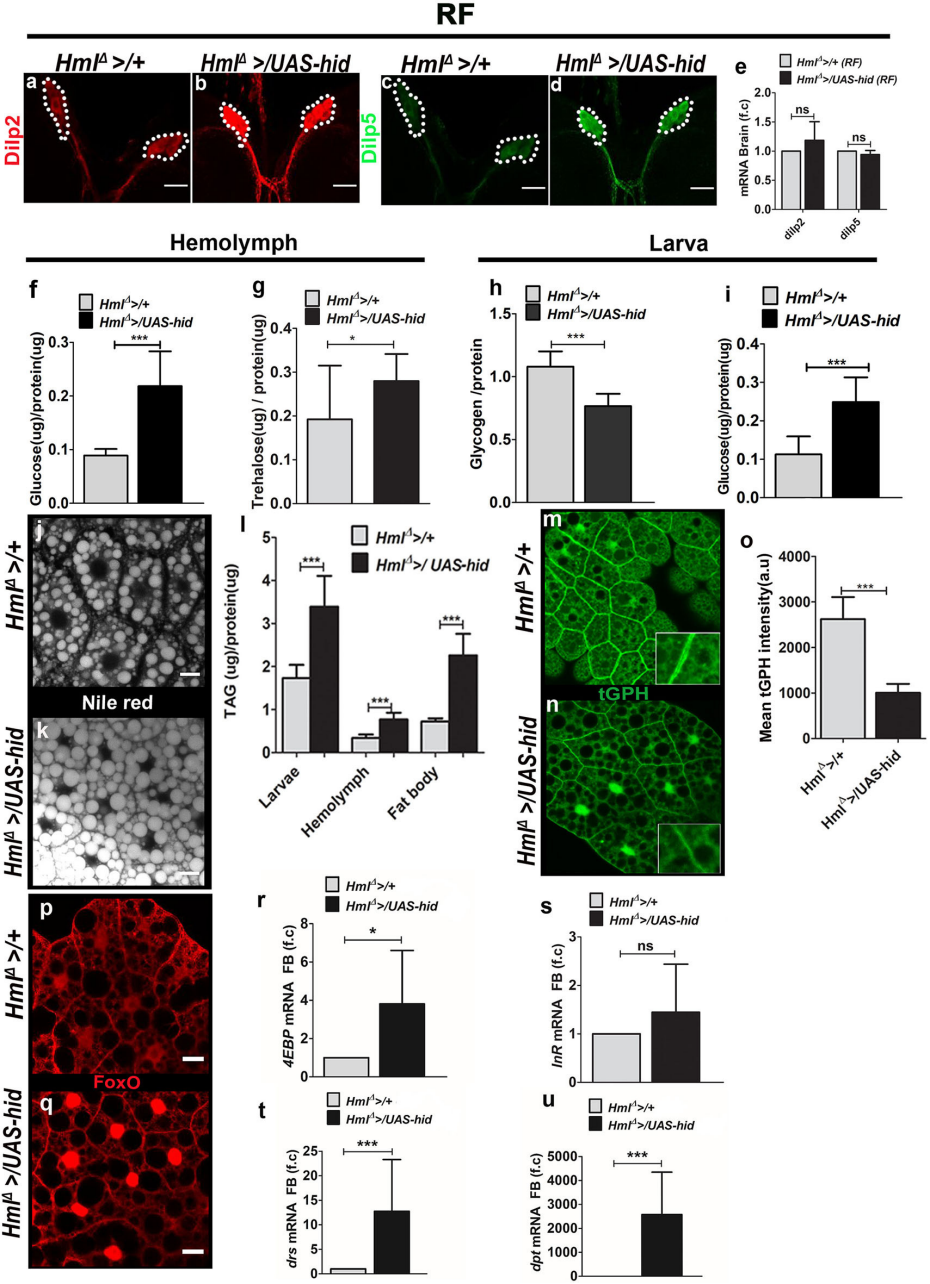
Ablation of Hml^+^ plasmatocytes regulates fat body insulin sensitivity and inflammatory homeostasis. In **(a–d,j,k,p,q)**, scale bar = 20 μm. In **(e–i,l,o,r–u)** bar graphs represent mean ± standard deviation (SD). Statistical analysis, unpaired *t*-test, two-tailed in **(e–i,l,o,r–u)**. “n” is the total number of larvae analyzed, RF is regular food, FB is fat body, f.c is fold change, a.u is arbitrary units. **(a–e)** Dilp2 and Dilp5 analysis in feeding L3 larval brains. Immunostainings of **(a,b)** Dilp2 and **(c,d)** Dilp5 peptides. As compared with **(a,c)** control (*Hml*^Δ^ >*/*+*)*, **(b,d)** *Hml*^Δ^ >*/UAS-hid* larval brains reveal increased Dilp2 and Dilp5 peptide expression in insulin-producing cells (IPCs). **(e)** qPCR analysis for *dilp2* and *dilp5* in brain tissue of feeding L3 larvae does not show any change in their relative mRNA expression. Relative fold change is represented and statistical analysis was performed on **C**_**t**_values (dilp2; *Hml*^Δ^ >*/*+, *n* = 70, 4 ± 0.32; *Hml*^Δ^ >*/UAS-hid, n* = 80, 3.77 ± 0.71 and dilp5; *Hml*^Δ^ >*/*+, *n* = 70, 9.25 ± 0.18; *Hml*^Δ^ >*/UAS-hid, n* = 80, 9.34 ± 0.29). **(f)** Hemolymph glucose levels. *Hml*^Δ^ >*/*+, *n* = 78, 0.09 ± 0.01 and *Hml*^Δ^ >*/UAS-hid n* = 78, 0.22 ± 0.06 (***p-value < 0.0001). **(g)** Hemolymph trehalose levels. *Hml*^Δ^ >*/*+, *n* = 90, 0.19 ± 0.10 and *Hml*^Δ^ >*/UAS-hid, n* = 72, 0.28 ± 0.06 (*p-value = 0.0335). **(h)** Whole-larvae glycogen levels. *Hml*^Δ^ >*/*+, *n* = 6, 1.08 ± 0.12 and *Hml*^Δ^ >*UAS hid, n* = 6, 0.77 ± 0.1 (***p-value = 0.0006). **(i)** Whole-larvae glucose levels. *Hml*^Δ^ >*/*+, *n* = 39, 0.11 ± 0.05 and *Hml*^Δ^ >*/UAS-hid, n* = 39, 0.25 ± 0.06 (***p-value < 0.0001). **(j,k)** Neutral lipid staining (Nile red) in fat bodies of **(j)** *Hml*^Δ^ >*/*+ and **(k)** *Hml*^Δ^ >*UAS-hid*. Compared with control, **(j)** *Hml*^Δ^ >*/*+, **(k)** *Hml*^Δ^ >*UAS-hid* fat bodies show more lipid droplets. **(l)** TAG levels measurement in whole larvae (*Hml*^Δ^ >*/*+, *n* = 36, 1.7 ± 0.3 and *Hml*^Δ^ >*UAS hid, n* = 36, 3.4 ± 0.7, ***p-value = 0.0005), hemolymph (*Hml*^Δ^ >*/*+, *n* = 60, 0.34 ± 0.08 and *Hml*^Δ^ >*/UAS-hid, n* = 66, 0.77 ± 0.16, ***p-value < 0.0001) and fat body (*Hml*^Δ^ >*/*+, *n* = 45, 0.7 ± 0.07 and *Hml*^Δ^ >*/UAS-hid, n* = 40, 2.25 ± 0.5, ***p-value < 0.0001). **(m–o)** tGPH expression in **(m,n)** fat bodies of feeding L3 larvae from **(m)** control (*Hml*^Δ^ >*/*+) and **(n)** *Hml*^Δ^ >*/UAS-hid* backgrounds shows reduced tGPH expression in *Hml*^Δ^ >*/UAS-hid* condition. **(o)** Quantification of mean tGPH intensities in *Hml*^Δ^ >*/*+ (*n* = 25, 2621 ± 486.2) and *Hml*^Δ^ >*/UAS-hid* (*n* = 25, 1005 ± 196, ***p-value = 0.0001). **(p,q)** FoxO immunostaining in fat bodies of feeding L3larvae from **(p)** control (*Hml*^Δ^ >*/*+) and **(q)** *Hml*^Δ^<*/UAS-hid* backgrounds. As compared with **(p)** control, FoxO is nuclear localized in fat bodies of *Hml*^Δ^<*/UAS-hid* animals. **(r,s)** Fat body analysis of FoxO target genes. **(r)** *4EBP* and **(s)** *InR* m*RNA* expression. *4EBP* is upregulated in *Hml*^Δ^ >*/UAS-hid* condition. Relative fold change is represented and statistical analysis was performed on **C**_**t**_values (*4EBP*: *Hml*^Δ^ >*/*+, *n* = 60, 2.53 ± 0.91; *Hml*^Δ^ >*/UAS-hid, n* = 60, 1.11 ± 0.45; *p-value = 0.0233 and *InR*; *Hml*^Δ^ >*/*+, *n* = 60, 10.15 ± 0.93; *Hml*^Δ^ >*/UAS-hid, n* = 60, 9.99 ± 1.31). **(t,u)** Fat body analysis of inflammatory pathway target genes. **(t)** *drs* and **(u)** *dpt* m*RNA* expression is significantly upregulated in *Hml*^Δ^ >*/UAS-hid* condition. Relative fold change is represented and statistical analysis was performed on **C**_**t**_values (*drs*: *Hml*^Δ^ >*/*+, *n* = 60, 5.40 ± 0.64; *Hml*^Δ^ >*/UAS-hid, n* = 60, 2.29 ± 1.28; ***p-value = 0.0002 and *dpt*: *Hml*^Δ^ >*/*+, *n* = 60, 16.79 ± 1.16; *Hml*^Δ^ >*/UAS-hid, n* = 60, 5.78 ± 0.69; ***p-value < 0.0001).

Insulin signaling is a central regulator of animal growth (37). To understand the underlying regulation by blood cells in coordinating systemic growth, we undertook an in-depth analysis of insulin signaling in *Hml*^Δ^ >*/UAS-hid* animals. We first assessed production and expression of different Dilp genes from the insulin producing cells (IPCs) of feeding L3 larvae. Dilp release from the IPCs is dependent on feeding state of the animal. As long as larvae are feeding, Dilps are produced and released from the IPCs. In non-feeding state or in conditions of nutritional deprivation such as starvation, Dilp release is inhibited leading to their accumulation in the IPCs (37). We conducted immunohistochemical and quantitative m*RNA* analysis of Dilp 2 and 5. Compared with control larvae, *Hml*^Δ^ >*/UAS-hid* animals showed increased Dilp 2 and Dilp 5 peptide expression in the IPCs (Figures 2a–d, Supplementary Figures 3a,b). To test whether the increase was a consequence of heightened synthesis of Dilp 2 and 5, qRT-PCR analysis was done for *Dilp 2* and *5* m*RNA* levels in feeding L3 larval brain tissues (Figure 2e). There was no increase in the levels of mRNA suggesting that the increase observed in Dilp 2 and Dilp 5 peptide expression was most likely a consequence of its accumulation or abrogated release. Blocking Dilp release or its accumulation in the brain IPCs is associated with hyperglycemia, which is a characteristic of reduced insulin signaling. Therefore, we tested readouts of glucose homeostasis, measuring circulating levels of glucose and trehalose as well as whole-animal glucose and glycogen and TAG levels. These biochemical assays were performed on feeding L3 larvae from control and *Hml*^Δ^ >*/UAS-hid* backgrounds. As reported in conditions of reduced Dilp release, in *Hml*^Δ^ >*/UAS-hid* animals, a significant increase of glucose and trehalose was observed in the circulating hemolymph (Figures 2f,g). Whole-animal glycogen was reduced (Figure 2h), whereas glucose levels were upregulated (Figure 2i). Further, increased levels of TAG were observed in whole larvae, circulating hemolymph, and fat body (Figure 2l). Neutral lipids detected via Nile red staining also confirmed increased TAG accumulation in *Hml*^Δ^ >*/UAS-hid* fat bodies (Figures 2j,k). Lipid droplet size in these fat bodies was comparatively larger than seen in control conditions (Figures 2j,k).

Consistent with the biochemical analysis, assessment of downstream readouts of insulin signaling in the fat body also revealed a reduction in insulin signaling. Fat body glucose levels were reduced (Supplementary Figure 3c). Expression levels of tGPH, a membrane-associated GFP expression marker whose fluorescence is an indicator of insulin-dependent PI3K activity in living cells (38, 39), showed a reduction in *Hml*^Δ^ >*/UAS-hid* larval fat bodies (Figures 2m–o). Insulin-mediated repression of FoxO nuclear localization and signaling (40) was also affected in *Hml*^Δ^ >*/UAS-hid* larvae. FoxO protein (detected using antibodies against it), which was detected primarily in the cytoplasm of control fat body tissues, in *Hml*^Δ^ >*/UAS-hid* larval fat bodies, was nuclear localized (Figures 2p,q). Subsequently, we also checked for the mRNA levels of FoxO targets *4EBP, InR*, and *tobi* in the fat body by isolating RNA followed by qRT-PCR from control and *Hml*^Δ^ >*/UAS-hid* conditions. This detected a significant upregulation in the levels of *4EBP* (Figure 2r) and mild upregulation of *InR* (Figure 2s) in *Hml*^Δ^ >*/UAS-hid* conditions compared with controls. Together, these data show that the adult growth retardation was a consequence of reduced fat body insulin signaling in *Hml*^Δ^ >*/UAS-hid* conditions. To determine if these changes were a result of the defect in fat body Akt signaling, we assessed levels of phosphorylated Ser505-Akt. Although immunohistochemical analysis did show a reduction in pAkt level, a quantitative analysis of its expression with respect to total Akt revealed only a minor difference in *Hml*^Δ^ >*/UAS-hid* animals (Supplementary Figures 3d–f). This suggests either Akt independence (41) or phosphorylation at other sites on Akt, which are more sensitive indicators of its function (29) and remains to be addressed.

A strong connection between activation of fat body innate immune signaling leading to loss of insulin sensitivity (42, 43) and animal growth defect is well-established (29). Given the growth defect seen upon loss of immune cells, we asked if immune cell loss led to any changes in fat body innate immune signaling. For this, we investigated Toll and Imd innate immune signaling pathways in fat bodies in control and *Hml*^Δ^ >*/UAS-hid* conditions. Expression analysis of *drosomycin (drs)*, a Toll pathway target gene, and *diptericin (dpt)*, an Imd pathway target gene, was undertaken by isolating RNA from the fat body of *Hml*^Δ^ >*/UAS-hid* and control larvae. Compared with expression in controls, *Hml*^Δ^ >*/UAS-hid* conditioned fat bodies showed a strong upregulation of both *drs* (Figure 2t) and *dpt* (Figure 2u). This indicated a robust activation of innate immune signaling pathways in the fat body on the loss of Hml^+^ immune cells, implying a role for these cells in moderating activation of inflammatory pathways in the fat body.

### Hemocytes Control Tolerance to High-Sucrose Diet

**Figure 3:**
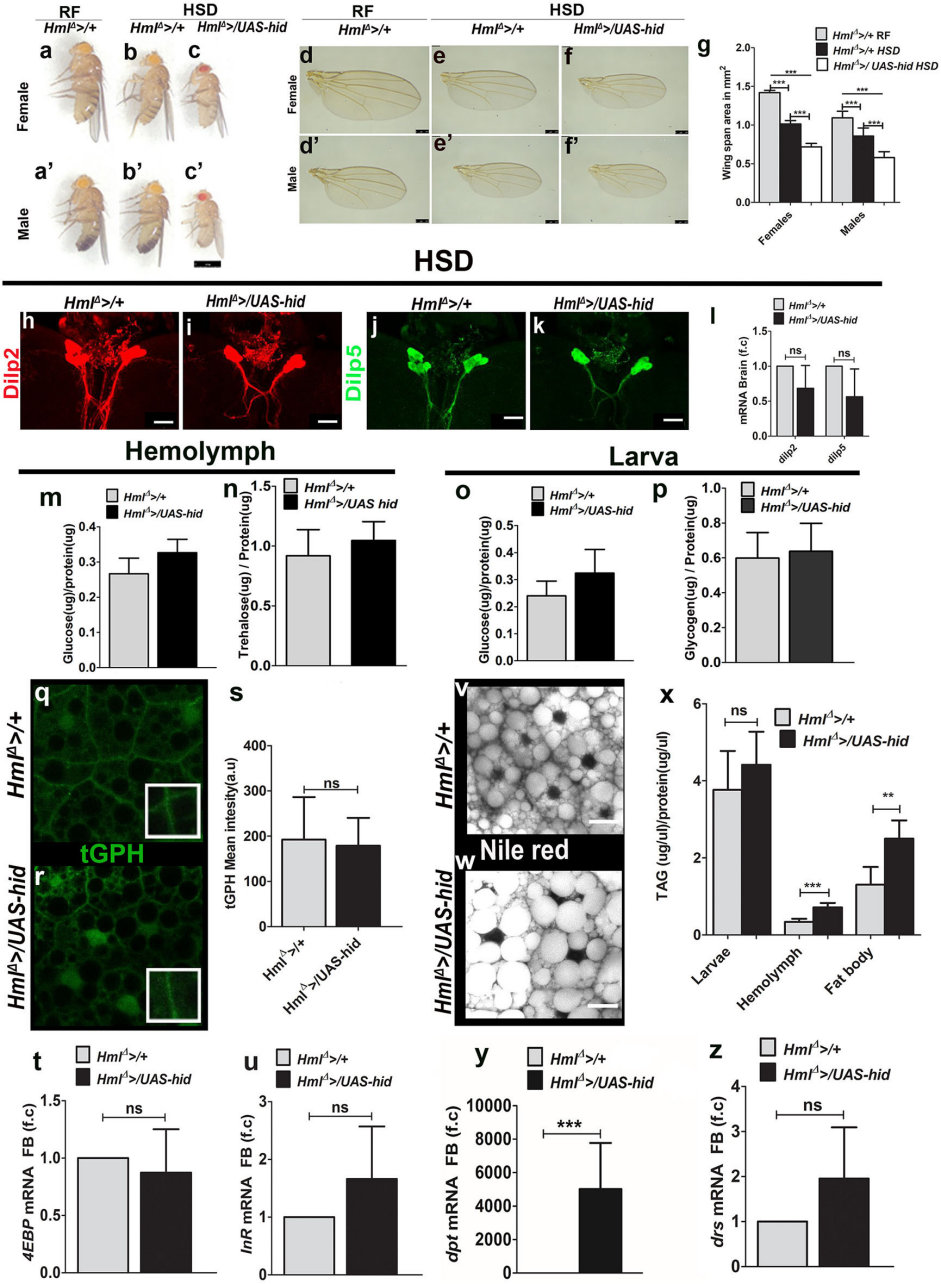
Immune cells are necessary for tolerance to high dietary sugar–induced metabolic stress. Scale bars in **(a–c′)** = 1 mm, **(d–f′)** = 250 μm, and **(h–k,v,w)** = 20 μm. Bar graphs in **(g,l,m–p,s–u)**, and **(x–z)** represent mean ± standard deviation (SD). Statistical analysis in **(g,l,m–p,s–u,x–z)** is unpaired *t*-test, two-tailed and two-way ANOVA comparison in **(g)**. “n” is total number of larvae analyzed, HSD is high sugar diet, RF is regular food, FB is fat body, f.c is fold change, a.u is arbitrary units. **(a–g)** Blood cell ablation worsens HSD induced adult growth defect. Compared with controls (*Hml*^Δ^ >*/*+) reared on **(a,a′)** RF, **(b,b′)** HSD induces a growth retardation which is **(c,c′)** worsened in *Hml*^Δ^ >*/UAS-hid* HSD animals. **(d–g)** Representative images showing adult size defect. In comparison with **(d,d′)** *Hml*^Δ^ >*/*+ on RF, **(e,e′)** *Hml*^Δ^ >*/*+ on HSD show reduction in wing sizes which is further reduced in **(f,f′)** *Hml*^Δ^ >*/UAS-hid* HSD adults. **(g)** Quantification of wing span areas in: *Hml*^Δ^ >*/*+ on RF (female *n* = 100, 1.4 ± 0.03, male *n* = 100, 1.1 ± 0.09) *Hml*^Δ^ >*/*+ on HSD (female *n* = 100, 1.0 ± 0.04, ***p-value < 0.0001, male *n* = 100, 0.856 ± 0.1, ***p-value < 0.0001) and *Hml*^Δ^ >*/UAS-hid* on HSD (female *n* = 100, 0.7 ± 0.04, ***p-value < 0.0001 and male *n* = 100, 0.6 ± 0.08, ***p-value < 0.0001). Two-way ANOVA analysis was performed (females ***p-value < 0.0001 and males ***p-value < 0.0001). **(h–l)** Dilp2 and Dilp5 analysis in feeding L3 larval brains raised on HSD. Immunostainings of **(h,i)** Dilp2 and **(j,k)** Dilp5 peptides in **(h,j)** control (*Hml*^Δ^ >*/*+) and **(i,k)** *Hml*^Δ^ >*/UAS-hid* larval brains do not show any change in their peptide expression in insulin-producing cells (IPCs). **(l)** qPCR analysis for *dilp2* and *dilp5* in brain tissue of feeding L3 larvae does not show any change in their relative mRNA expression. Relative fold change is represented and statistical analysis was performed on **C**_**t**_values (*dilp2*; *Hml*^Δ^ >*/*+, HSD, *n* = 100, 4.86 ± 1.27; *Hml*^Δ^ >*/UAS-hid*, HSD, *n* = 100, 5.92 ± 1.46 and *dilp5*; *Hml*^Δ^ >*/*+, HSD, *n* = 100, 9.15 ± 2.13; *Hml*^Δ^ >*/UAS-hid*, HSD, *n* = 100, 10.66 ± 2.48). **(m)** Hemolymph glucose levels. *Hml*^Δ^ >*/*+, HSD, *n* = 42, 0.27 ± 0.04 and *Hml*^Δ^ >*/UAS-hid*, HSD, *n* = 42, 0.33 ± 0.04 (*p-value = 0.0189). **(n)** Hemolymph trehalose levels. *Hml*^Δ^ >*/*+, HSD, *n* = 36, 0.92 ± 0.22, and *Hml*^Δ^ >*/UAS-hid*, HSD, *n* = 36, 0.28 ± 0.2. **(o)** Whole-larvae glucose levels. *Hml*^Δ^ >*/*+, HSD *n* = 18, 0.24 ± 0.05 and *Hml*^Δ^ >*/UAS-hid n* = 18, 0.32 ± 0.09. **(p)** Whole-larvae glycogen levels. *Hml*^Δ^ >*/*+, HSD, *n* = 13, 0.6 ± 0.15 and *Hml*^Δ^ >*/UAS-hid* HSD, *n* = 13, 0.64 ± 0.16. **(q–s)** tGPH expression in **(q,r)** fat bodies of feeding L3 larvae from **(q)** *Hml*^Δ^ >*/*+, HSD controls and **(r)** *Hml*^Δ^ >*/UAS-hid*, HSD backgrounds showed no change in tGPH expression. **(s)** Quantification of mean tGPH intensities in *Hml*^Δ^ >*/*+ on HSD (*n* = 15, 192 ± 94) and *Hml*^Δ^ >*/UAS-hid*, on HSD (*n* = 20, 178 ± 61). **(t,u)** Fat body analysis of FoxO target genes. **(t)** *4EBP* and **(u)** *InR* m*RNA* expression show no difference. Relative fold change is represented and statistical analysis was performed on **C**_**t**_values. (*4EBP*: *Hml*^Δ^ >*/*+, HSD, *n* = 80, 0.29 ± 0.65; *Hml*^Δ^ >*/UAS-hid*, HSD, *n* = 80, 0.61 ± 1.18 and *InR*: *Hml*^Δ^ >*/*+, HSD, *n* = 80, 8.97 ± 1.34; *Hml*^Δ^ >*/UAS-hid*, HSD, *n* = 80, 8.43 ± 1.91). **(v,w)** Neutral lipid (Nile red) staining in fat bodies of larvae raised on HSD. Compared with **(v)** *Hml*^Δ^ >*/*+, on HSD **(w)** *Hml*^Δ^ >*/UAS-hid* HSD animals show increased number of bigger lipid droplets. **(x)** TAG levels measurements in whole larvae (*Hml*^Δ^ >*/*+, HSD, *n* = 24, 3.8 ± 1 and *Hml*^Δ^ >*UAS hid*, HSD, *n* = 24, 4.4 ± 0.86), hemolymph (*Hml*^Δ^ >*/*+, HSD, *n* = 60, 0.34 ± 0.08 and *Hml*^Δ^ >*/UAS-hid*, HSD, *n* = 60, 0.7 ± 0.1, ***p-value < 0.0001), and fat body (*Hml*^Δ^ >*/*+, HSD *n* = 30, 1.304 ± 0.5 and *Hml*^Δ^ >*/UAS-hid*, HSD, *n* = 30, 2.5 ± 0.5, **p-value = 0.0013). **(y,z)** qPCR analysis of **(y)** *dpt* and **(z)** *drs* m*RNA* expression in fat body tissue of feeding L3 larvae raised on HSD. Relative fold change is represented and statistical analysis was performed on **C**_**t**_values (*dpt: Hml*^Δ^ >*/*+ HSD *n* = 80, 17.96 ± 0.88; *Hml*^Δ^ >*/UAS-hid*, HSD, *n* = 80, 5.96 ± 0.75; ***p-value < 0.0001 and *drs: Hml*^Δ^ >*/*+, HSD, *n* = 80, 8.13 ± 0.48; *Hml*^Δ^ >*/UAS-hid*, HSD, *n* = 80, 7.12 ± 1.11) *indicates signifcant *p* values.

It is known that larvae fed on a high-sucrose diet develop as smaller adults (21). High sugar stress also corresponds with a reduction in immune cell numbers (44). The sugar stress–induced growth defect is a consequence of reduction in fat body insulin signaling (39, 45). *Hml*^Δ^ >*/UAS-hid* larvae with reduced Hml^+^ immune cells demonstrated a similar growth defect, reduced insulin signaling, elevated fat body inflammation, and hyperglycemia, all of which are characteristic signs of insulin resistance. Hence, it was important to investigate how loss of Hml^+^ immune cells influenced tolerance to additional high sugar dietary stress. To address this, larvae were raised on 25% sucrose, referred to as high-sucrose diet (HSD) in the text. Compared with the growth defect seen in control animals fed on HSD, *Hml*^Δ^ >*/UAS-hid* animals on HSD showed further growth retardation (Figures 3a–g). The growth retardation was also detected when these animals were raised on diet with elevated fructose (Supplementary Figures 4a–c). This result showed that the growth defect was a consequence of high dietary sugar induced stress and was not limited to sucrose-enriched diet, suggesting a growth promoting or stress relieving function of Hml^+^ innate immune cells in conditions of high dietary sugar.

To address if any changes in insulin signaling could explain the worsening of growth in *Hml*^Δ^ >*/UAS-hid* HSD animals, we analyzed different components of insulin signaling, as described in the previous section. Estimation of expression of Dilp2 and 5 peptides (Figures 3h–k, Supplementary Figures 4d,e) and their m*RNA* levels in the larval brain IPCs (Figure 3l) showed no change between *Hml*^Δ^ >*/UAS-hid* and controls. Biochemical analysis revealed a mild increase in larval hemolymph glucose in *Hml*^Δ^ >*/UAS-hid* HSD animals (Figure 3m), but trehalose levels remained comparable with control HSD larvae (Figure 3n). Whole-animal glucose and glycogen failed to detect any changes in their levels (Figures 3o,p). Readouts of fat body insulin signaling did not reveal any difference either. Membrane tGPH expression (Figures 3q–s), fat body glucose levels (Supplementary Figure 4f), phosphorylated S505-Akt levels (Supplementary Figures 4g,h,k), nuclear localization of FoxO protein (Supplementary Figures 4i,j), and expression of FoxO target genes (Figures 3t,u and Supplementary Figure 4l) remained comparable between control HSD and *Hml*^Δ^ >*/UAS-hid* HSD larvae. Thus, the growth defect seen in *Hml*^Δ^ >*/UAS-hid* HSD animals was not a consequence of any dramatic change in glucose homeostasis or insulin signaling.

Immune cell ablation, however, impacted the TAG levels in these animals. Although overall larval TAG levels remained comparable, hemolymph and fat body TAG levels showed a significant increase in *Hml*^Δ^ >*/UAS-hid* HSD larvae (Figure 3x). Nile red staining of fat bodies confirmed the increase in the TAG levels as well (Figures 3v,w). The lipid droplet sizes detected in *Hml*^Δ^ >*/UAS-hid* HSD fat bodies were comparatively larger than seen in the control tissues (Figures 3v,w). Together, these results showed a defect in the fat body lipid metabolism in the *Hml*^Δ^ >*/UAS-hid* HSD larvae.

We next assessed the status of fat body innate immune signaling in response to immune cell ablation in HSD condition. Interestingly, *Hml*^Δ^ >*/UAS-hid* animals showed upregulation of Imd target gene, *dpt* (Figure 3y), whereas expression of Toll target gene, *drs*, remained comparable with control HSD fat bodies (Figure 3z). This suggested specific activation of the Imd pathway on loss of immune cells in HSD condition and was unlike regular dietary state, where loss of Hml^+^ cells led to dramatic upregulation of both Toll and Imd signaling in the fat body. In conditions of dietary excess, such specific modulation of fat body inflammatory signaling (46) and influence on tolerance to metabolic toxicity (47) is reported. Our findings support this notion and implicate Hml^+^ immune cells in moderating this specificity.

### Increasing the Number of Activated Blood Cells in *Drosophila* Larvae Rescues the HSD Induced Growth Defect

**Figure 4:**
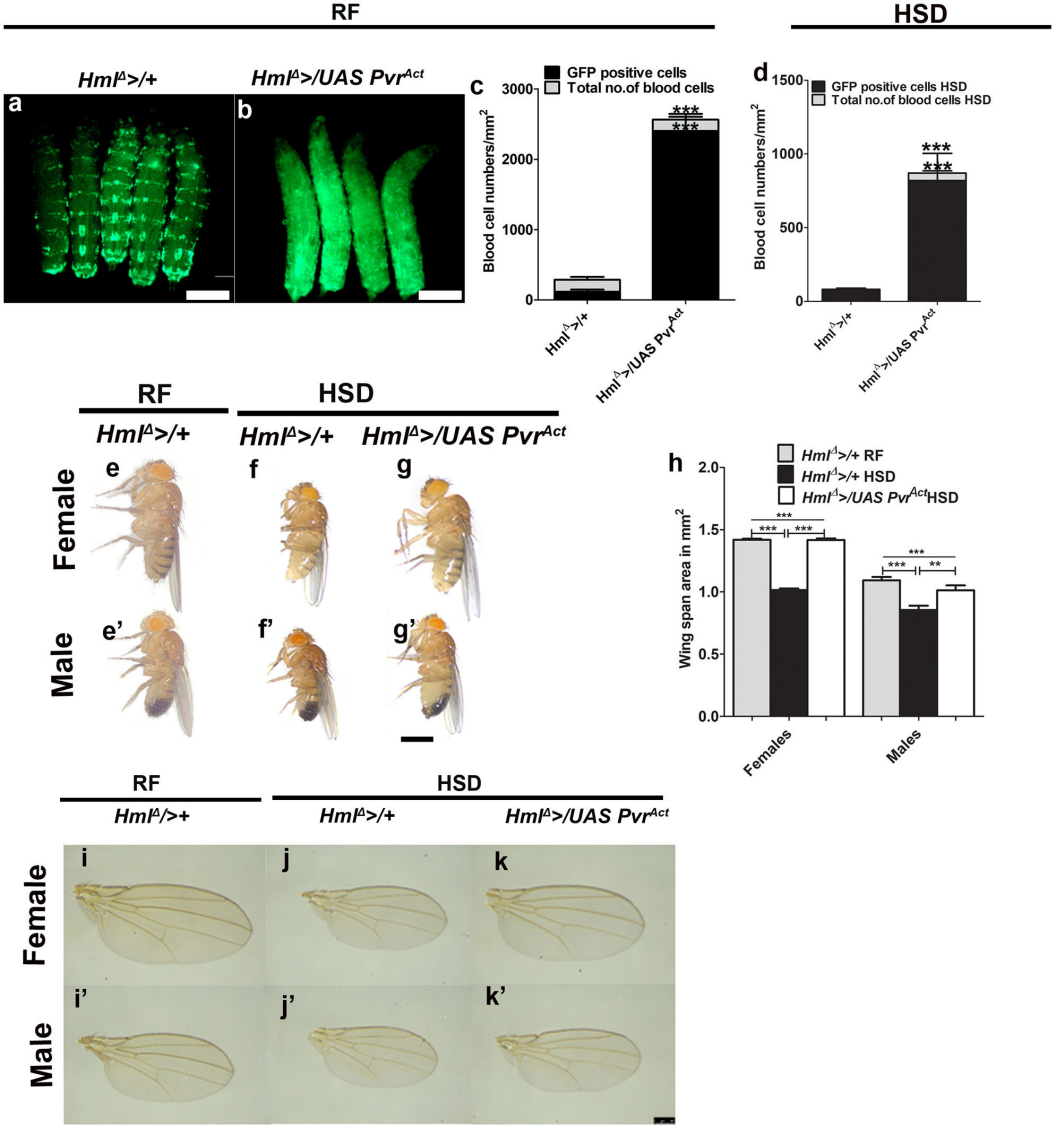
Increased immune cell activity restores adult growth defect seen in high dietary sugar condition. In **(a,b,e–g′)**, scale bar = 1 mm, **(i–k′)** = 250 μm. In **(c,d,h)**, bar graphs show mean ± standard deviation (SD) and statistical analysis in these panels is unpaired *t*-test, two-tailed. “*n*” is the total number of larvae analyzed, RF indicates regular food, HSD indicates high sugar diet. **(a–d)** Expressing *Pvr*^*Act*^ in blood cells (Hml, green) causes an expansion in their numbers. Compared with **(a)** control (*Hml*^Δ^ >*UAS-GFP*) larvae, **(b)** *Hml*^Δ^ >*/UAS Pvr*^*Act*^ animals have more blood cells. Blood cell quantifications on **(c)** RF and **(d)** HSD. **(c)** *Hml*^Δ^ >*/*+ on RF (total number of blood cells: *n* = 10, 202.39 ± 26 and Hml^+^ cells, *n* = 10, 117.7 ± 28.3) and *Hml*^Δ^ >*/UAS Pvr*^*Act*^ on RF (total number of cells: *n* = 10, 2563.15 ± 226.47, ***p-value < 0.0001 and Hml^+^ cells, *n* = 10, 2403.7 ± 200.04, ***p-value < 0.0001). **(d)** *Hml*^Δ^ >*/*+ on HSD (total number of blood cells, *n* = 10, 81.4 ± 16 and Hml^+^ cells, *n* = 10, 70 ± 17) and *Hml*^Δ^ >*/UAS Pvr*^*Act*^ on HSD (total number of blood cells, *n* = 10, 871 ± 189, ***p-value < 0.0001 and Hml^+^ cells, *n* = 10, 819 ± 185, ***p-value < 0.0001). **(e–g′)** Expressing *Pvr*^*act*^ in blood cells restores HSD induced adult growth defect. Compared with *Hml*^Δ^ >*/*+ reared on **(e,e′)** RF**, (f,f′)** HSD induced growth retardation is **(g,g′)** restored in *Hml*^Δ^ >*/UAS Pvr*^*Act*^ HSD animals. See quantifications in **(h). (h)** Quantification of wing span areas. *Hml*^Δ^ >*/*+ reared on RF (female = 1.4 ± 0.03, *n* = 100; male = 1.1 ± 0.08, *n* = 100), *Hml*^Δ^ >*/*+ on HSD (female = 1 ± 0.04, *n* = 100, ***p-value < 0.0001 in comparison with *Hml*^Δ^ >*/*+ reared on RF, male 0.9 ± 0.11, *n* = 100, ***p-value < 0.0001 in comparison with *Hml*^Δ^ >*/*+ reared on RF) and *Hml*^Δ^ >*/UAS Pvr*^*Act*^ on HSD (female = 1.4 ± 0.05, *n* = 100, ***p-value < 0.0001 in comparison with *Hml*^Δ^ >*/*+ reared HSD, male 1.01 ± 0.12, *n* = 100, **p-value = 0.0064 in comparison with *Hml*^Δ^ >*/*+ reared on HSD). Two-way ANOVA comparison was performed (females ***p-value < 0.0001 and males ***p-value < 0.0001). **(l–k′)** Representative wing images of adult flies showing growth restoration of *Hml*^Δ^ >*/UAS Pvr*^*Act*^ adults on HSD. **(i–j′)** *Hml*^Δ^ >*/*+on **(i,i′)** RF and **(j,j′)** on HSD and **(k,k′)** *Hml*^Δ^ >*/UAS Pvr*^*Act*^ on HSD.

We next assessed the outcome of increasing immune cell numbers on organismal metabolic state and growth homeostasis. For this, we over-expressed a constitutively active version of PDGF/VEGF-like receptor *(Pvr*^*act*^*)* in blood cells using *Hml*^Δ^ > as the driver (*Hml*^Δ^ >*/UAS Pvr*^*Act*^) (48). This genetic manipulation resulted in a dramatic increase in immune cell numbers and specifically of Hml^+^ cells (Figures 4a–c, Supplementary Figures 5a–b′′′). This manipulation leads to the expansion of immune cells that are characteristically similar to the invasive macrophages (49). In regular food conditions, adult flies from this genetic background did not show any effect on growth phenotype and were comparable in size with control adult flies (Supplementary Figures 5g–k). This result suggested that although animal growth is sensitive to loss of immune cells (Figures 1, 2), a mere increase in the immune cell numbers did not result in a concomitant increase in animal sizes. These data suggest that immune cells are not directly involved in scaling of animal sizes. Consistent with this notion, insulin signaling remained unaffected in *Hml*^Δ^ >*/UAS Pvr*^*Act*^ animals (Supplementary Figures 5c–f,l–v).

Next, we explored the influence of increased immune cell numbers on dietary sugar–induced growth defect and tolerance. Compared with immune cell numbers of controls on HSD, *Hml*^Δ^ >*/UAS Pvr*^*Act*^ raised on HSD had significantly higher immune cell numbers (Figure 4d), but not as dramatic as seen in regular condition (compared with Figure 4c). The proportion of Hml^+^ immune cells was specifically increased (Figure 4c). Interestingly, the growth defect seen in HSD animals was dramatically restored in *Hml*^Δ^ >*/UAS Pvr*^*Act*^ HSD genetic background (Figures 4e–h). Their sizes were comparable with sizes seen for controls raised on regular dietary state (Figures 4g,g′ compared with Figures 4e,e′). A similar trend of increased immune cell numbers was also evident with overexpression of wild-type Pvr in Hml^+^ (*Hml*^Δ^ >*/Pvr*^*WT*^) blood cells (Supplementary Figures 6a,b,d). These animals were also significantly larger than HSD controls (Supplementary Figures 6e–g), but smaller than *Hml*^Δ^ >*/UAS Pvr*^*Act*^ HSD animals (compare Figure 4h with Supplementary Figure 6g). The difference in growth restoration between *Hml*^Δ^ >*/UAS Pvr*^*Act*^ and *Hml*^Δ^ >*/Pvr*^*WT*^ may stem from the extent of increased numbers of activated immune cells, which is much higher in *Hml*^Δ^ >*/UAS Pvr*^*Act*^ as opposed to *Hml*^Δ^ >*/Pvr*^*WT*^ (Figure 4c compared with Supplementary Figure 6d). Importantly, increasing immune cell numbers using other genetic manipulations did not lead to a growth restoration phenotype. Expression of a temperature-sensitive form of shibire, shi^ts^ in Hml^+^ cells (*Hml*^Δ^ >*/UAS shi*^*ts*^), which also causes a comparable expansion of Hml^+^ immune cells as seen in *Hml*^Δ^ >*/Pvr*^*WT*^ (Supplementary Figures 6a,c,d), was insufficient to recover the growth defect of HSD. Contrastingly, *Hml*^Δ^ >*/UAS shi*^*ts*^ HSD animals were smaller and demonstrated growth retardation (Supplementary Figures 6h–j). These data suggested immune cell state as a key component in growth control. We conclude that immune cell activity is linked to systemic growth control as opposed to only their numbers being a regulator for growth.

We further tested other dietary sugar–induced stress, such as fructose and glucose, and observed that different sugars had varying effects on growth. Compared with the growth of control animals on sucrose-rich diet, the growth reduction was severe in high-glucose diet, whereas fructose-rich diet showed a mild growth defect (Supplementary Figures 7a,b); *Hml*^Δ^ >*/UAS Pvr*^*Act*^ animals were able to restore growth in all conditions. This was not seen in *Hml*^Δ^ >*/UAS shi*^*ts*^ animals. *Hml*^Δ^ >*/UAS Pvr*^*Act*^ condition restored growth in every dietary condition, but at differential capacities. This trend was not evident in *Hml*^Δ^ >*/UAS shi*^*ts*^ animals (Supplementary Figures 7c–r). This result further strengthened the importance of immune cell states in moderating dietary stress–induced growth defect.

**Figure 5:**
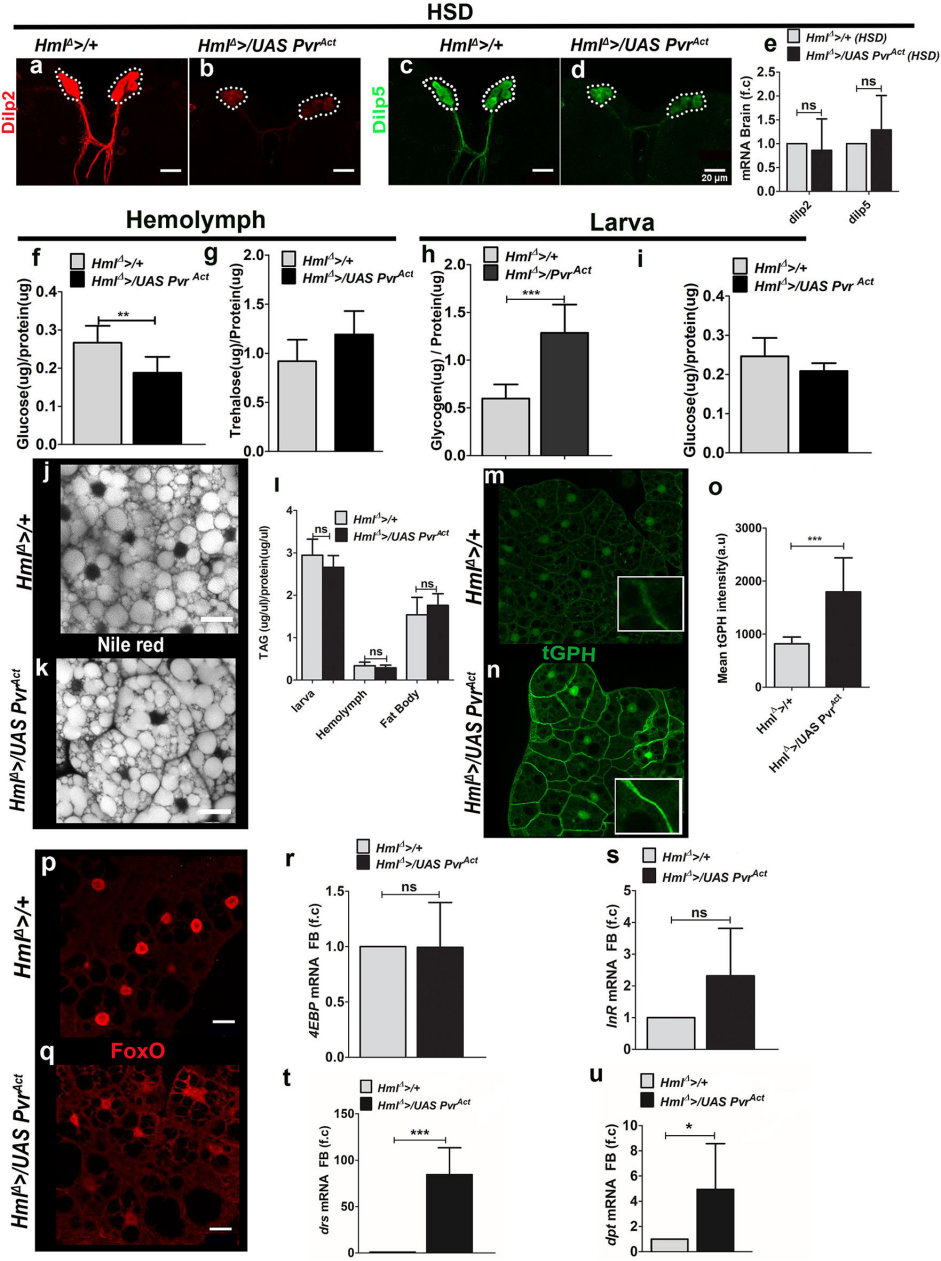
Increased immune cell activity improves fat body insulin sensitivity and tolerance on HSD condition. In **(a–d,m,n,p,q)**, scale bar = 20 μm. In **(e–i,l,o,r-u)** bar graphs show mean ± standard deviation (SD) and statistical analysis applied in these panels is unpaired *t-*test, two-tailed. “*n*” is the total number of larvae analyzed, HSD indicates high-sugar diet, a.u is arbitrary units, FB is fat body. **(a–e)** Dilp2 and Dilp5 expression analysis in feeding L3 larvae reared on HSD. **(a–d)** Immunostaining of **(a,b)** Dilp2 and **(c,d)** Dilp5 peptide expression in insulin-producing cells (IPCs) of **(a,c)** control (*Hml*^Δ^ >*/*+) on HSD and **(b,d)** *Hml*^Δ^ >*/UAS Pvr*^*Act*^ HSD brains show reduced Dilp2 and Dilp5 levels in *Hml*^Δ^ >*/UAS Pvr*^*Act*^ condition. **(e)** Relative quantification of *dilp2* and *dilp5*m*RNA* levels showed no change. Relative fold change is represented and statistical analysis was done using **C**_**t**_values (*dilp2*; *Hml*^Δ^ >*/*+, HSD, *n* = 100, 4.86 ± 1.27; *Hml*^Δ^ >*/UAS Pvr*^*Act*^, HSD, *n* = 100, 5.66 ± 1.52 and *dilp5*; *Hml*^Δ^ >*/*+, HSD, *n* = 100, 9.15 ± 2.13; *Hml*^Δ^ >*/UAS Pvr*^*Act*^, HSD, *n* = 100, 9.91 ± 1.11). **(f)** Hemolymph glucose levels (*Hml*^Δ^ >*/*+, HSD, *n* = 42, 0.27 ± 0.04 and *Hml*^Δ^ >*/UAS Pvr*^*Act*^, HSD, *n* = 42, 0.19 ± 0.4, **p-value = 0.0051). **(g)** Hemolymph trehalose levels (*Hml*^Δ^ > */*+ HSD, *n* = 36, 0.92 ± 0.22 and *Hml*^Δ^ >*/UAS Pvr*^*Act*^, HSD, *n* = 48, 1.2 ± 0.2). **(h)** Whole-larvae glycogen levels (*Hml*^Δ^ >*/*+, HSD, *n* = 13, 0.6 ± 0.15 and *Hml*^Δ^ >*/UAS Pvr*^*Act*^, HSD, *n* = 15, 1.3 ± 0.3, ***p-value < 0.0001). **(i)** Whole-larvae glucose levels. (*Hml*^Δ^ */*+, HSD, *n* = 18, 0.25 ± 0.05 and *Hml*^Δ^ >*/UAS Pvr*^*Act*^, HSD, *n* = 18, 0.21 ± 0.02). **(j,k)** Neutral lipid (Nile red) staining in fat bodies of **(j)** *Hml*^Δ^ >*/*+ on HSD and **(k)** *Hml*^Δ^ >*/UAS Pvr*^*Act*^ on HSD revealed no change in lipid levels. **(l)** TAG measurements in whole larvae (*Hml*^Δ^ >*/*+ HSD *n* = 18, 3 ± 0.4 and *Hml*^Δ^ >*/UAS Pvr*^*Act*^ *n* = 18, 2.7 ± 0.3), hemolymph (*Hml*^Δ^ >*/*+ HSD *n* = 60, 0.34 ± 0.08 and *Hml*^Δ^ >*/UAS Pvr*^*Act*^ HSD *n* = 60, 0.3 ± 0.06) and fat body (*Hml*^Δ^ >+ HSD *n* = 30, 1.54 ± 0.41 and *Hml*^Δ^ >*/UAS Pvr*^*Act*^ HSD *n* = 30, 1.76 ± 0.3). **(m–o)** tGPH expression in fat bodies of feeding L3 larvae reared on HSD. Compared with **(m)** HSD control (*Hml*^Δ^ >*/*+), **(n)** *Hml*^Δ^ >*/UAS Pvr*^*Act*^, HSD animals show restored membrane tGPH expression. **(o)** Mean tGPH intensity quantifications of *Hml*^Δ^ >*/*+ on HSD, *n* = 15, 818 ± 126 and *Hml*^Δ^ >*/UAS Pvr*^*Act*^ on HSD, *n* = 15, 1796 ± 640 (***p-value < 0.0001). **(p,q)** FoxO immunostaining in fat bodies of feeding L3 larvae reared on HSD. Compared with **(p)** nuclear FoxO expression in control (*Hml*^Δ^ >*/*+) on HSD, **(q)** *Hml*^Δ^ >*/UAS Pvr*^*Act*^, HSD animals show lesser levels in the nucleus. **(r,s)** Fat body analysis of FoxO target genes. **(r)** *4EBP* and **(s)** *InR* m*RNA* expression. Relative fold change is represented and statistical analysis was done using **C**_**t**_values (*4EBP*: *Hml*^Δ^ >*/*+, HSD, *n* = 80, 0.29 ± 0.65 and *Hml*^Δ^ >*/UAS Pvr*^*Act*^, HSD, *n* = 80, 0.39 ± 0.35 and (*InR*: *Hml*^Δ^ >*/*+, HSD, *n* = 80, 8.97 ± 1.34 and *Hml*^Δ^ >*/UAS Pvr*^*Act*^, HSD, *n* = 80, 7.98 ± 1.13). **(t,u)** qPCR analysis of **(t)** *drs* and **(u)** *dpt* m*RNA* expression in fat body tissue of feeding L3 larvae raised on HSD. Relative fold change is represented and statistical analysis was performed on **C**_**t**_ values (*drs*: *Hml*^Δ^ >*/*+, HSD, *n* = 80, 8.13 ± 0.48 and *Hml*^Δ^ >*/UAS Pvr*^*Act*^, HSD, *n* = 80, 1.81 ± 0.88; ***p-value < 0.0001 and *dpt*; *Hml*^Δ^ >*/*+, HSD, *n* = 80, 17.96 ± 0.88 and *Hml*^Δ^ >*/UAS Pvr*^*Act*^, HSD, *n* = 80, 15.74 ± 0.75 *p-value 0.0313).

We assessed the effect of *Pvr*^*Act*^ expression in hemocytes on peripheral insulin and inflammatory signaling in HSD condition and observed restoration of certain features of insulin resistance. The accumulation of Dilp2 and Dilp5 peptides normally seen in larval IPCs in control HSD animals was not observed in *Hml*^Δ^ >*/UAS Pvr*^*Act*^ HSD larval brain IPCs (Figures 5a–d, Supplementary Figures 8a,b). This was a not a consequence of reduction in *Dilp2* and *Dilp5* m*RNA* levels (Figure 5e). Biochemical analysis of circulating larval hemolymph glucose and trehalose revealed a reduction in glucose levels, whereas trehalose remained unchanged (Figures 5f,g). Whole-animal glycogen was comparatively higher in *Hml*^Δ^ >*/UAS Pvr*^*Act*^ HSD animals as compared with control groups (Figure 5h). However, whole-animal glucose levels remained unchanged (Figure 5i). These data suggested an improvement in circulating glucose and whole-animal glycogen levels in *Hml*^Δ^ >*/UAS Pvr*^*Act*^ HSD larvae. Lipid measurements, however, showed no change and remained comparable with control HSD conditions (Figures 5j–l).

Readouts of peripheral fat body insulin signaling revealed restoration of some of its features. Of these, membrane tGPH levels (Figures 5m–o) and FoxO localization (Figures 5p,q) were restored in *Hml*^Δ^ >*/UAS Pvr*^*Act*^ HSD larvae. Changes in the expression of FoxO target genes, *4EBP, InR*, and *tobi*, were not detected (Figures 5r,s, Supplementary Figure 8g). pAKT levels also did not increase (Supplementary Figures 8d–f). Examining Toll and Imd pathway targets revealed an unexpected upregulation of *drs* (Figure 5t) in *Hml*^Δ^ >*/UAS Pvr*^*Act*^ HSD animals, whereas *dpt*, the Imd pathway target gene, remained unchanged (Figure 5u). This was unlike the *Hml*^Δ^ >*/UAS hid* HSD animals where a dramatic upregulation of *dpt* was evident without any change in *drs* expression.

## Discussion

Much of our understanding of systemic control of animal growth is generally limited to endocrine organs. In *Drosophila*, the fat body (24), gut (15), and brain are highlighted as predominant nutrient sensors regulating growth (21). Because of nutrient sensing and signaling functions performed by these organs, they have been the primary focus of investigations on growth regulation. The formation of blood cells is also an energy-consuming process and has a metabolic cost on the animal. Immune cells are highly sensitive to nutrient modulation (18, 50). However, the physiological relevance of their increased sensitivity to dietary changes and impact on growth, if any, is poorly understood. Our study demonstrates the influence of immune cells on coordinating systemic metabolism and animal growth. In this regard, two important points emerge from this work: (1) immune cell states rather than their number is an important parameter in growth regulation and (2) immune cells systemically coordinate growth via the regulation of fat body inflammation and insulin signaling. These functions of blood cells support the attainment of proper adult size both in homeostasis and in conditions of sugar excess. While we focus on immune cell–fat body cross-talk, our results do not rule out any direct communication between immune cells and the brain or other organs in coordinating growth. Overall, this study positions innate immune cells as a novel player in organismal metabolic homeostasis and growth control regulation.

### Immune Cell/Fat Body Cross-Talk in Animal Growth Control

We find that in homeostatic conditions, modulating immune cell numbers in larval life impacts overall organismal metabolic state and animal growth. The loss of immune cells dramatically reduces adult growth. The metabolic and biochemical assays in these animals resembles features of systemic insulin insensitivity (51). This includes increased circulating TAGs, circulating glucose/trehalose levels with reduced whole-animal glycogen levels. Specifically, fat body metabolism, innate immune signaling, and insulin sensitivity are also affected. Past and recent findings have highlighted immune cell–mediated regulation of fat body immune activation and metabolic homeostasis by secreted factors like psidin (52), Upd3 (53), and adenosine (30). These interactions between immune cells and the fat body allow nutrient allocation to regulate animal growth in nutrient overload conditions (53) or in response to infection for an effective immune response (30, 52). Our study suggests a similar cross-talk between immune cells and the fat body in the maintenance of metabolic homeostasis. We posit that immune cell activity modulates fat body insulin signaling, which is central to organismal growth control. Loss of Hml^+^ immune cells corresponds with robust activation of fat body Toll and Imd signaling, indicating increased inflammation. This positions Hml^+^ cells as key regulators of fat body inflammatory homeostasis. Persistent activation of Toll (29) and Imd signaling pathways in the fat body (26, 28) leads to insulin insensitivity, metabolic dysregulation, and growth defect (54). Recently published data by Shin et al. (53) have highlighted a similar role for Hml^+^ cells in systemic control of animal growth by regulating fat body Jak/Stat signaling. Our data strengthen this notion and proposes a model where anti-inflammatory inputs by immune cells function on controlling fat body innate immune homeostasis and insulin sensitivity, thereby contributing to animal growth control (43). In addition, any change of Dilp 2 and Dilp5 production or secretion from the larval brain IPCs on immune cell manipulation cannot be ruled out. This can also lead to a reduction in circulating Dilps and reduced systemic insulin signaling. Whether this is mediated by the fat body (24) or by blood cells directly requires further validation.

However, in conditions of nutrient excess (HSD), the modulation of immune cell numbers on systemic metabolism, fat body inflammation, and its insulin sensitivity does not correlate with the findings in regular dietary conditions. These data are unexpected and indicate that cross-talk between blood cells and the fat body is more complex. Ablation of Hml^+^ blood cells further worsened the adult sizes without differences in insulin sensitivity. Fat body insulin signaling and overall glucose homeostasis were not different from what is observed in control animals raised on HSD. Interestingly, this resulted in preferential activation of the Imd pathway in the fat body, without any change in Toll signaling. On the other hand, increasing the numbers of activated immune cells (*Hml*^Δ^ >*Pvr*^*Act*^) relieved the symptoms of metabolic stress induced by HSD. Here, signatures of insulin resistance or fat body insulin sensitivity revealed only partial recovery, but the extent of growth restoration observed in these animals was comparable with wild types raised on a regular diet. In these growth-restored animals, a specific activation of fat body Toll signaling was evident. This is possible as activated immune cells are capable of secreting spaztle, the Toll ligand, and drive systemic Toll activation (55, 56). A similar influence of innate immune signaling on growth in nutrient overload is supported by the published literature. Peptidoglycan recognition proteins (PGRPs) are known to activate either Toll or Imd pathways, and their modulation in the fat body differentially influences animal growth. PGRP-SB2 activates the Imd pathway, and its loss in the fat body increases growth and survival on HSD. Although PGRP-SC2 negatively regulates the Imd pathway and positively regulates the Toll pathway, its loss in the fat body reduces animal size on HSD (27).

Together, these data reveal that growth is differentially controlled in dietary excess as opposed to homeostasis and is supported by the independence of animal growth from insulin signaling seen in HSD *Hml*^Δ^ >*hid* and *Hml*^Δ^ >*Pvr*^*Act*^ conditions. Our data also suggest an additional growth-promoting axis independent of insulin signaling regulated by blood cell activity. Here, we propose a model where immune cells function to balance fat body innate immune activation. This supports fat body nutrient reallocation toward the promotion of animal growth and prevents metabolic toxicity. The additional immune cell/fat body inflammatory axis compensates for reduced fat body insulin signaling in HSD conditions.

### Immune Cell State as the Driver of Growth Control

Cross-talk between the fat body and immune cells could either be dependent on immune cell numbers (3) or activity. Our findings reveal the importance of immune cell state to sustain metabolism and growth capacity. The expression of a constitutively active form of Pvr in immune cells resulted in the activation of invasive plasmatocytes (6, 49) and improved tolerance to dietary excess. Increasing immune cell numbers by overexpression of *shi*^*ts*^ did not ameliorate the HSD growth defect. On the contrary, *Hml*^Δ^ >*/UAS shi*^*ts*^ animals were smaller. Loss of shibire function blocks exocytosis (57, 58); therefore, shibire lacking blood cells are functionally inert, unlike *Pvr*^*Act*^-expressing hemocytes, which are more active. Immune cell states are reflective of their internal metabolic activity (25) and signaling capacities (52, 53, 56, 59). The elevated metabolic state of active immune cells may therefore provide an animal with additional means to metabolize nutrients (especially when in excess) and does not require increased immune cell number with inert metabolic states. The immune cell states may be a reflection of changes in immune heterogeneity (25, 53) or internal metabolic states as seen in development (25) or in conditions of stress (20).

### A Model of Temporal Control of Immune Cell Function in Regulation of Animal Growth

Immune cell function in growth homeostasis is temporally controlled and is required early, before the 3rd instar. This is inferred from the pronounced deficit in adult growth after loss of immune cells in early stages of larval development and not if immune cells are depleted post 3rd instar. Although larval growth is unchanged after immune cell depletion, subtler changes in larval sizes or weight cannot be ruled out. The reduced adult size may stem from a reduction in cell growth and proliferation (60, 61). This is supported by analysis of cell densities in wing imaginal discs of wandering 3rd instar *Hml*^Δ^ >*hid* larvae, which showed a reduction in wing disc cell densities with increased spacing between cells (Supplementary Figures 2i–k). Suppression of insulin receptor signaling late in development affects body and organ size as opposed to its early role in developmental timing. This transition from the control of developmental timing to growth occurs early in 3rd instar, when larvae reach critical size, post which development can be completed in the absence of food. Consequently, critical size is a key stage in insect development that is established in early 3rd instar larvae and sets the lower limit of final adult size. The mechanisms that measure the critical size and the organs involved in this process are largely unclear. Fat body, imaginal discs are the only predicted critical-size sensing organs thus far. Based on the early immune function apparent from this study, we hypothesize an involvement of immune cells in the assessment of larval critical weight, supported by the evidence of cross-talk in this work. The implication of immune cell/fat body interaction early in larval life could be relevant in the establishment of developmental switches or programs that time the acquisition of critical weight and the temporal shift in insulin signaling to allow growth in non-feeding larval and pupal stages (60). The comparable metabolic and growth phenotypes seen in animals with reduced Inr activity late in larval life with animals depleted for immune cells are in agreement with this hypothesis.

## Conclusion

The fat body functions to integrate the physiological state of the animal and determine allocation of resources in a context-dependent manner (23). Much like this tissue, immune cells, in addition to their role in sensing infection, are also effective sensors of changes in nutrient levels (3, 20, 62). However, unlike the fat body which is fixed in location, immune cells are mobile and highly dynamic. Their behavior and localization change rapidly in contexts of stress or nutrient modulation (3, 30). Our findings clearly implicate immune cells as central players coordinating global metabolic homeostasis and growth control along with the fat body. This integrates both the cellular and humoral component of the innate immune system, which may have evolved for efficient allocation of resources during infections but is also coopted in development to orchestrate systemic metabolism and growth. Metabolic disorders like diabetes, obesity, and fatty liver in mammals (62) are associated with heightened inflammation and altered immune responses. This exemplifies a connection between immune cells, altered metabolic homeostasis, and disease progression; however, its relevance in development regulation like growth remains unaddressed. Future investigations will be necessary to probe the temporal nature of this cross-talk to reveal mechanistic insights underlying developmental paradigms operating in animal growth and physiology.

## Methods

### *Drosophila* Husbandry, Stocks, Genetics, and Food

The following *Drosophila* stocks were used in this study: *w*^1118^ (wild-type), *Hml*^Δ^ >*UAS-Gfp* (S.Sinenko), *domeMESO-GFP*; *hml-dsRed* (Utpal Banerjee), *Hml*^Δ^ >*Gfp;tub gal 80*^*ts*^, *Hml*^Δ^ >*Gfp;tGPH, UAS-hid/CyoGFP, UAS-Pvr*^*Act*^ and *UAS-Shi*^*ts*^ (63), *UAS Pvr* (BL58998), and *Hml*^Δ^*gal4* (BL30141). All fly stocks were reared on the standard BDSC corn meal agar food medium with yeast supplementation (referred to as the regular food in the article) at 25°C incubator unless specified. The specific composition of the regular food (RF) for 1 L is corn flour, 80 g; d-glucose, 20 g; sugar, 40 g; agar, 8 g; yeast powder, 15 g; propionic acid, 4 mL; Tego (methyl parahydroxy benzoate), 1 g (5 mL ethanol); and orthophosphoric acid, 0.6 mL. For high-sugar diet (HSD), the regular food composition was modified by supplementing the food with 25% sucrose (20 g in 100 mL of standard medium) whereas the composition of the other ingredients remained unchanged. For high-fructose diet (HFD), the regular food composition was modified by supplementing the food with 25% d-fructose (25 g in 100 mL of standard medium) while the composition of the other ingredients remained unchanged. For high-glucose diet (HGD), the regular food composition was modified by supplementing the food with 25% d-glucose (23 g in 100 mL of standard medium) whereas the composition of the other ingredients remained unchanged. All genetic crosses were set up at 25 °C and then transferred to 29°C where they were grown until analysis either as larvae or as adults.

### Embryo Collection

Embryo collections were done for 4–6 h at 25°C. This was followed strictly for all experimental crosses. For HSD experiments, the embryos were collected on RF at 25°C after which 40–50 embryos were carefully transferred to HSD and reared at 29°C until analysis. Temperature-sensitive experiments with *tub gal80*^*ts*^ were carefully monitored to maintain timings for shifting them from a non-permissive (18°C) to permissive (29 °C) temperature. Specifically, for *Hml*^Δ^ >*UAS-hid;tub gal80*^*ts*^, the embryo collection was conducted at 25°C and shifted to 18°C where they were grown until mid-2^nd^ instar larvae followed by shift to 29°C incubator. For *Hml*^Δ^ >*UAS shi*^*ts*^ experiments after embryo collection, the animals were grown at 29°C until analysis.

### Quantification of Adult Growth Phenotype

All adult flies were scored for their body sizes 2 days after eclosion. The animals were scored first by comparing their body sizes against the control *Hml*^Δ^ >*/w*^1118^ reared either on regular food or on HSD. The wingspan area of the adult animals was also scored to quantify for growth (64). The wings of flies of interest were plucked, mounted on a slide, and imaged using a bright-field microscope. Female and male wings were mounted separately and analyzed separately. The wingspan areas were calculated using Fiji software. Briefly, the circumference of the wing is marked and area is measured. Only one wing per animal has been analyzed. In every experiment, we analyzed 12–15 animals. This was done with a minimum of 10 repeats.

The wingspan area quantification for genotypes *UAS hid* and *UAS Pvr*^*Act*^ on RF were carried out together in multiple batches. Hence, the controls are the same in Figure 1h and Supplementary Figure 5k.

### Immunostaining, Immunohistochemistry, and Fluorescence Quantification

For rabbit pAKT (1:400) and rabbit FoxO (1:500) staining, fat bodies from feeding 3^rd^ instar larvae were dissected in 1 × PBS, fixed in PBS containing 4% formaldehyde for 20 min at room temperature, and washed in PBS containing 0.1% Triton X-100 (PBT). Tissues were then blocked for 2 h in 0.3% PBT containing 5% NGS. Primary antibodies were incubated overnight at 4°C and secondary antibodies for 2 h at room temperature.

For Dilp 2 (1:400) and Dilp 5 (1:800) stainings, brains were dissected from feeding L3 larvae in 1 × PBS. They were fixed in 1 × PBS containing 4% formaldehyde for 20 min at room temperature, and extensively washed in 1 × PBS containing 0.3% Triton X-100 (PBT). Tissues were then blocked for 2 h in 0.3% PBT containing 5% NGS. Primary antibodies were incubated overnight at 4°C and secondary antibodies for 2 h at room temperature. To quantify Dilp2 and Dilp5 levels, confocal Z series of the IPCs were obtained using a 2-μm step size and identical laser power and scan settings. Fiji software was used to generate sum-intensity 3D projections of the Z stacks (16-bit scanned images) and to measure total fluorescent intensity across the IPCs.

For staining circulating blood cells, 3^rd^ instar larvae were bled on Teflon-coated slides (Immuno-Cell no. 2015 C 30) followed by staining protocol as previously described.

The following secondary antibodies have been used in the study at 1:500 dilution: FITC and Cy3 (Jackson Immuno Research Laboratories). Phalloidin (Sigma-Aldrich no. 94072) was used at 1:100 dilutions to stain cell morphologies and nuclei were visualized using DAPI. Samples were mounted with Vectashield (Vector Laboratories) or 70% glycerol.

### Imaging

Immunostained images, blood cell images, and wing Disc images were acquired using Olympus FV3000 confocal microscopy system under a × 20 air or × 40 oil-immersion objective or × 60 oil-immersion objective. Bright-field and larval fluorescence images were obtained on Leica fluorescence stereomicroscope.

### Hemocyte Isolation and Quantification

Total blood cells including circulating hemocytes and sessile pool resistant hemocytes were isolated. For this, larvae where mechanically brushed to release the sessile pool resident hemocytes into circulation, and after dissection, the cells still adhering to cuticle were scraped with the forceps as per published protocol (65).

Circulating cell numbers obtained were quantified per larvae. For each genotype, a minimum of 10 larvae were analyzed. Five images per well covering the field of view were obtained under constant magnification. The hemocytes in these views were counted manually to score for DAPI-positive (representing total blood cells), Hml-positive, and Hml-negative cells. The counts are represented as blood cell numbers per square millimeter (53).

### Quantification of tGPH

tGPH intensity is quantified using ImageJ software as previously described in (39). Briefly, fat bodies of feeding L3 larvae were imaged using confocal microscopy and average fluorescence was measured in 25 random squared areas (10 × 30 pixels), each covering part of the plasma membrane in different cells.

### Nile Red Staining

For lipid droplet staining, wandering L3 larvae were dissected in 1 × PBS and fixed in 4% formaldehyde in 1 × PBS for 20 min at room temperature. Tissues were then rinsed twice with 1 × PBS, incubated for 30 min in a 1:1000 dilution with 70% glycerol of 0.02% Nile red (Sigma—Cat. no. N3013). The tissues were mounted in 70% glycerol with DAPI.

### Metabolite Measurements

Glucose, trehalose, and glycogen measurements were done in feeding 3^rd^ instar larvae. Triglyceride measurements were done in wandering 3^rd^ instar larvae.

Glucose and TAG assays were conducted in extracts made from whole larva (1 sample = 3 larvae) and fat body (1 sample = fat body from 5 larvae). These tissues were homogenized in 100 μL of 1 × PBS using GENETIX Bead Beater to obtain the extract for glucose or TAG analysis. For hemolymph extracts, bleeds from six larvae was collected in 100 μL of 1 × PBS and centrifuged at 1000 rpm to remove blood cells. The extracted samples were heat inactivated at 70°C for 10 min and then centrifuged at 1000 rpm. The supernatant was collected and subjected to glucose analysis using Sigma GOD-POD kit (GAGO20) or TAG analysis using the Sigma Triglycerides assay reagents (T2449 and F6428) (66).

For hemolymph trehalose assays, we adapted a previously published protocol (66). Briefly, larval hemolymph from six animals was collected in 25 μL of ice-cold 1 × PBS and centrifuged at 5,000 rpm for 5 min to remove blood cells. 10 μL of sample was incubated in 25 μL of 0.25 M sodium carbonate at 95°C for 2 h in a thermal cycler, then cooled to room temperature followed by additions of 8 μL of 1 M acetic acid and 66 μL of 0.25 M sodium acetate (pH 5.2) as the digestion buffer. 1 μL of porcine trehalase (Sigma T8778) was added to 40 μL of this mixture and incubated at 37°C overnight. The resulting glucose was analyzed and normalized to protein levels.

The trehalose measurements for genotypes *UAS hid* and *UAS Pvr*^*Act*^ on RF and HSD were carried out together in multiple batches. Hence, the controls are same in Figures 5p, 2g (RF) and Figures 3n, 5g (HSD).

Whole-larvae glycogen assay was conducted as per the instructions provided along with Glycogen Assay Kit (MAK016) (67). For this one, whole larva was homogenized in 100 μL of 1 × PBS. This extract was used for the assay and the amounts were normalized to total protein levels in the same sample.

The glycogen measurements for genotypes *UAS hid* and *UAS Pvr*^*Act*^ on RF and HSD were carried out together in multiple batches. Hence, Figures 5o, 2h (RF) and Figures 3p, 5h (HSD) share the same controls.

Protein estimation was undertaken using the Thermo Scientific BCA protein assay kit (Cat. no. 23225) and Varioskan LUX Multimode Microplate Reader using skanit software was used to quantify all metabolites.

### Cell Density Quantification

Wing disc cell density was quantified by counting the total number of nuclei stained with DAPI in five random regions (430 μm^2^ area each) of the wing imaginal disc. This was done for five different wing discs (68).

### RNA Extraction and RT-PCR Analysis

RNA extractions from larval fat bodies(20 larvae) and brain (35 larvae) tissues were performed as previously published (69). Briefly, RNA from these samples was extracted using Trizol reagent (Ambion by Life Technologies Cat no. 15596). For RT-PCR, RNA samples were treated with DNase I (Thermo Scientific) and converted to cDNA with SuperScript II (Invitrogen). qPCR was performed using the SYBR Green PCR Master Mix (Applied Biosystems), in the C-1000 Touch Thermal Cycler (BIO-RAD CFX384 Real-Time System) in 384-well plates (Applied Biosystems). At least three biological replicates were used for statistical analysis. The following primers were used to perform qPCR:

**Table:** List of qPCR primers

***Gene***	***Primer***	***Reference***
*InR*	*F−5′-ACTGAACCTCTCGTCAAGGC-3′*	*(70)*
	*R−5′-GAACCCTCCACGCACTTACA-3′*	
*tobi*	*F−5′-CCACCAAGCGAGACATTTACC-3′*	*(70)*
	*R−5′-GAGCGGCGTAGTCCATCAC-3′*	
*4EBP*	*F−5′-CCAGGAAGGTTGTCATCTCG-3′*	*(70)*
	*R−5′-CCAGGAGTGGTGGAGTAGAGG-3′*	
*dpt*	*F−5′-ACCGCAGTACCCACTCAATC-3′*	*Designed using NCBI primer blast*
	*R−5′-CCCAAGTGCTGTCCATATCC-3′*	
*drs*	*F−5′-GTACTTGTTCGCCCTCTTCG-3′*	*Designed using NCBI primer blast*
	*R−5′-CTTGCACACACGACGACAG-3′*	
*rp49*	*F−5′-CGGATCGATATGCTAAGCTGT-3′*	*Gifted by Dr. Raghu Padinjat Lab, NCBS*
	*R−5′-GCGCTTGTTCGATCCGTA-3′*	

The mRNA quantifications for genotypes *UAS hid* and *UAS Pvr*^*Act*^ on HSD were carried out together in multiple batches. Hence, Figures 3t,u,y,z, 5r,s,t,u, Supplementary Figures 4l, **8g** share the same controls.

### Protein Extraction and Western Blot Analysis

Tissues (fat bodies from 20 larvae and brains from 35 larvae) were homogenized with the help of stainless steel beads (Qiagen; 69989) in an EZ-Lyser bead beater (Genetix). Protein extraction was carried out as previously published (69). Protein estimations were done using Bradford Reagent (Sigma B6916). 10% SDS-PAGE and Western blots were performed using standard methods (69). The following antibodies were used: primary antibodies—anti-pAkt (1:1000; rabbit; CST 4054; Ser505), anti-Akt (1:1000; rabbit; CST 9272), and anti-β-tubulin (1:3000; rabbit; Abcam ab6046) and secondary antibodies—anti-rabbit IgG, HRP-linked (CST 7074). Chemiluminescent reagent Western Bright Quantum (Advansta r-03026-c50) was used for detection by iBright FL1000 (Invitrogen). For measurements of pAKT/AKT ratios, the band mean intensities of pAkt and Akt were quantified with the help of Fiji (ImageJ) and corrected for background levels, followed by calculating their ratios from which the fold change was obtained.

The immunoblot quantifications for genotypes *UAS*-*hid* and *UAS Pvr*^*Act*^ on HSD were carried out together in multiple batches. Hence, Supplementary Figures 4k, 8f share the same control.

## Statistical Analysis

All statistical analysis was performed using Graph Pad Prism 5 and Microsoft Excel 2010. The means were analyzed using two-tailed, unpaired Student *t* test. Two-way ANOVA was performed between wing span areas of *Hml*^Δ^ >*/*+ RF, *Hml*^Δ^ >*/*+ HSD versus *Hml*^Δ^ >*/UAS-hid* RF, *Hml*^Δ^ >*/UAS-hid* HSD (Figure 3g) and *Hml*^Δ^ >*/*+ RF, *Hml*^Δ^ >*/*+ HSD versus *Hml*^Δ^ >*/UAS Pvr*^*Act*^ RF, *Hml*^Δ^ >*/UAS Pvr*^*Act*^ HSD (Figure 4h).

## Data Availability Statement

All datasets generated for this study are included in the article/Supplementary Material.

## Author Contributions

PP and TM conceptualized the project. PP, AT, and SM performed experiments, conducted the formal analysis, and contributed to the writing of the manuscript. TM contributed towards procuring funding to drive this research, overseen the project for its scientific, critical evaluation of the data and manuscript writing and editing. All authors contributed to the article and approved the submitted version.

## Conflict of Interest

The authors declare that the research was conducted in the absence of any commercial or financial relationships that could be construed as a potential conflict of interest.

## Supplementary Material

Supplementary Figure 1 In **(d–h****″****)**, scale bar = 20 μm. Bar graph in **(c)** shows mean ± standard deviation (SD). Statistical analysis applied in panel **c** is unpaired *t*-test, two-tailed in **(c)**. “*n*” is the total number of larvae analyzed. **(a–c)** *UAS-hid* genetic background does not show any blood cell defect. 3rd instar larval images depicting Hml^+^ blood cells (red, marked with RFP) in **(a)** control (*domeMESO-GFP; hml-dsRed/*+) and **(b)** *domeMESO-GFP; hml-dsRed/UAS-hid* are comparable. **(c)** Blood cell counts/mm^2^ in these backgrounds (*domeMESO-GFP; hml-dsRed/w1118, n* = 5, 166.5 ± 111.7 and *domeMESO-GFP; hml-dsRed/UAS-hid, n* = 5, 165.8 ± 92.7). **(d–i****″****)** Lineage analysis of *Hml*^Δ^*gal4* using GTRACE confirms blood-specific expression. RFP (real-time *Hml*^Δ^*gal4* expression) or GFP (lineage-based *Hml*^Δ^*gal4* expression) was not detected in **(d–d****″****)** brain, **(e–e****″****)** fat body, **(f–f****″****)** proventriculus, **(g–g****″****)** leg disc, **(h–h****″****)** wing disc. **(i–i****″****)** Both RFP and GFP expression is detected in blood cells.

Supplementary Figure 2 In **(b,c,d–e****′****)**, scale bar = 1 mm, **(f–g****′****)** = 250 μm, and **(i,j)** = 20 μm. In **(h**, **k)**, bar graphs show mean ± standard deviation (SD). Statistical analysis in **(h**, **k)** is unpaired *t*-test, two-tailed. “*n*” is the total number of larvae analyzed. **(a)** Shows the pictorial representation of the constitutive and temporal experiments that have been done using *Hml*^Δ^ > and *Hml*^Δ^ >*; tub gal80*^*ts*^, respectively. The red mark indicates the temporal expression of *UAS hid* in blood cells by shifting the larvae from permissive (18°C) to non-permissive temperature (29°C). **(b,c)** Temporal expression of *UAS-hid* in Hml^+^ blood cells (marked by GFP, green) causes a loss of blood cells. Compared with **(b)** control (*Hml*^Δ^ >*UAS-GFP; tub gal80*^*ts*^*/*+), **(c)** *Hml*^Δ^ >*UAS-GFP; tub gal80*^*ts*^*/ UAS hid* larvae show no GFP signal. **(d–h)** Temporal expression of *UAS-hid* in Hml^+^ blood cells did not alter adult growth. Representative adult **(d–e****′****)** fly and **(f–g****′****)** wing images in **(d, d****′****, f, f****′****)** control (*Hml*^Δ^ >*UAS-GFP; tub gal80*^*ts*^*/*+) and **(e,e****′****,g,g****′****)** *Hml*^Δ^ >*UAS-GFP; tub gal80*^*ts*^*/ UAS hid* show no growth reduction. **(h)** Wingspan area quantifications in *Hml*^Δ^ >*; tub gal80*^*ts*^*/*+ (female *n* = 100, 1.4 ± 0.1, male *n* = 100, 1.05 ± 0.07) and in *Hml*^Δ^ >*; tub gal80*^*ts*^*/UAS-hid* (female *n* = 100, 1.3 ± 0.1, male *n* = 100, 1.05 ± 0.09). **(i–k)** Reduced cell density in wing discs of *Hml*^Δ^ >*/UAS-hid* L3 larvae. Compared with cell density seen in wing discs of **(i)** control (*Hml*^Δ^ >*/*+), **(j)** *Hml*^Δ^ >*/UAS-hid* cell density is reduced. **(k)** Quantified by counting DAPI-positive cells (white). *Hml*^Δ^ >*/*+, *n* = 5, 11.24 ± 3, and *Hml*^Δ^ >*/UAS-hid, n* = 5, 4.80 ± 0.60 and (^**^*p*-value = 0.0017).

Supplementary Figure 3 In **(d,e)**, scale bar = 20 μm. In **(a–c**, **g)**, bar graphs show mean ± standard deviation (SD) and statistical analysis applied in these panels is unpaired *t*-test, two-tailed. “*n*” is the total number of larvae analyzed, FB is fat body, a.u is arbitrary units, f.c is fold change and RF indicates regular food. **(a)** Quantification of mean intensity of Dilp2 levels of images shown in Figures 2a,b. Control, *Hml*^Δ^ >*/*+ (*n* = 16, 204.5 ± 149.8) and *Hml*^Δ^ >*/UAS-hid* (*n* = 14, 1768.8 ± 794.2, ^***^*p*-value < 0.0001). **(b)** Quantification of mean intensity of Dilp5 levels of images shown in Figures 2c,d. Control, *Hml*^Δ^ >*/*+ (*n* = 16, 234.023 ± 321.72) and *Hml*^Δ^ >*/UAS-hid* (*n* = 13, 1395.4 ± 1303.9, ^**^*p*-value = 0.0019). **(c)** Fat body glucose levels. *Hml*^Δ^ >*/*+ (*n* = 65, 0.095 ± 0.017) and *Hml*^Δ^ >*/UAS-hid* (*n* = 65, 0.04 ± 0.02, ^***^*p*-value < 0.0001). **(d–f)** Fat body pAKT analysis. **(d,e)** Immunostaining of feeding L3 larval fat bodies with anti-pAKT antibody in **(d)** control, *Hml*^Δ^ >*/*+ and **(e)** *Hml*^Δ^ >*/UAS-hid* backgrounds show reduced pAKT levels in *Hml*^Δ^ >*/UAS-hid* condition. **(f)** Immunoblot analysis of pAkt/Akt ratio in fat bodies of feeding L3 larvae of control (*Hml*^Δ^ >*/*+) and *Hml*^Δ^ >*/UAS-hid* reveals a small difference (fold change ± SD mentioned in the blots). β-Tubulin was used as the internal loading control. **(g)** Relative fat body m*RNA* levels of *tobi*. Fold change is plotted, and statistical analysis was done using C_t_values (*Hml*^Δ^ >*/*+, *n* = 60,8.67 ± 3.57 and *Hml*^Δ^ >*/UAS-hid, n* = 60, 10.68 ± 0.75).

Supplementary Figure 4 In **(a–b****′****)**, scale bar = 250 μm and **(g–j)** is 20 μm. In **(c–f,l)**, bar graphs show mean ± standard deviation (SD) and statistical analysis applied for these panels is unpaired *t*-test, two tailed. “*n*” is the total number of larvae analyzed, FB is fat body, a.u is arbitrary units, f.c is fold change, HFD is high-fructose diet and HSD is high-sugar diet. **(a–c)** Loss of immune cells affects tolerance to high fructose diet. (**a–b****′**) Representative wing images of **(a,a****′****)** control (*Hml*^Δ^ >*/*+) on HFD and **(b,b****′****)**, *Hml*^Δ^ >*/UAS-hid* on HFD showing reduction in wing sizes of *Hml*^Δ^ >*/UAS-hid*, HFD animals. **(c)** Quantification of wingspan areas of *Hml*^Δ^ >*/*+ on HFD (female, *n* = 50, 1.4 ± 0.03, male *n* = 120, 1.1 ± 0.08) and *Hml*^Δ^ >*/UAS-hid* on HFD (female *n* = 120, 1.4 ± 0.06, ^***^*p*-value < 0.0001, compared with *Hml*^Δ^ >*/*+ HFD females, male *n* = 120, 1.1 ± 0.06, ^*^*p*-value <0.0333, compared with *Hml*^Δ^ >*/*+ males). **(d)** Quantification of mean intensity of Dilp2 expression of representative images shown in Figures 3h,i. Control, *Hml*^Δ^ >*/*+on HSD (*n* = 9, 2499 ± 561) and *Hml*^Δ^ >*/UAS-hid* on HSD (*n* = 8, 2681 ± 387.4). **(e)** Quantification of mean intensity of Dilp5expression of representative images shown in Figures 3j,k. Control, *Hml*^Δ^ >*/*+on HSD (*n* = 10, 1521 ± 425) and *Hml*^Δ^ >*/UAS-hid* on HSD (*n* = 7, 1463 ± 467). **(f)** Fat body glucose levels. *Hml*^Δ^ >*/*+ on HSD (*n* = 30, 0.021 ± 0.019) and *Hml*^Δ^ >*/UAS-hid* on HSD (*n* = 30, 0.02 ± 0.01). (**g,h**) pAKT immunostaining in fat bodies of feeding L3 larvae on HSD of **(g)** control (*Hml*^Δ^ >*/*+) and **(h)** *Hml*^Δ^ >*/UAS-hid* are comparable. **(i,j)** FoxO immunostaining in fat bodies of feeding L3 larvae on HSD of **(i)** control (*Hml*^Δ^ >*/*+) and **(j)** *Hml*^Δ^ >*/UAS-hid* show similar FoxO nuclear localization. **(k)** Immunoblot analysis of pAkt/Akt ratio in fat bodies of feeding L3 control (*Hml*^Δ^ >*/*+) and *Hml*^Δ^ >*/UAS-hid* larvae raised on HSD show no change. Fold change ± SD mentioned in the blots. β-Tubulin was used as the internal loading control. **(l)** Relative fat body m*RNA* levels of *tobi*. Fold change is plotted, and statistical analysis was done using C_t_ values (*Hml*^Δ^ >*/*+ on HSD, *n* = 80, 11.83 ± 1.32 and *Hml*^Δ^ >*/UAS-hid* on HSD *n* = 80, 11.69 ± 0.98).

Supplementary Figure 5 In **(a–f,l–n****′****,q–s****′****)**, and **(u–u****′****)** scale bar = 20 μm, **(g–h****′****)** scale bar = 1 mm, and **(i–j****′****)** scale bar = 250 μm. In **(k,l–p,t,v)**, bar graphs show mean ± standard deviation (SD) and in these panels statistical analysis applied is unpaired *t*-test, two tailed. “*n*” is the total number of larvae analyzed. a.u is arbitrary unit and RF is regular food. **(a–b****′′′****)** Characterization of blood cells in *Hml*^Δ^ >*UAS-GFP/UAS Pvr*^*Act*^ backgrounds. Compared with blood cells in **(a–a****′′′****)** control, *Hml*^Δ^ >*UAS-GFP*, **(b–b****′′′****)** *Hml*^Δ^ >*UAS-GFP/UAS Pvr*^*Act*^ larvae have increased numbers as evident from increased **(a,b)** DAPI, **(a****′****,b****′****)** Hml (*Hml*^Δ^ >*UAS-GFP)* and **(a****″****,b****″****)** phalloidin stainings. **(a****′′′****,b****′′′****)** Merge of all the channels. **(c–f)** Immunostainings of **(c,d)** Dilp2 and **(e,f)** Dilp5 in L3 feeding larval brain insulin-producing cells (IPCs). As compared with **(c,e)** control (*Hml*^Δ^ >*/*+) on RF, **(d,f)** *Hml*^Δ^ >*/UAS Pvr*^*Act*^ on RF, no change in Dilp2 and Dilp5 staining is detected. Quantification of mean intensities in **(l,m)**. **(g–k)** Expression of *UAS Pvr*^*Act*^ in Hml^+^ blood cells have not altered adult growth on RF. Representative adult **(g–h****′****)** fly and **(i–j****′****)** wing images in **(g,g****′****,i,i****′****)** control (*Hml*^Δ^ >*/*+) and **(h,h****′****,j,j****′****)** *Hml*^Δ^ >*/UAS Pvr*^*Act*^ show no growth modulation. **(k)** Quantifications of wing span areas of control *Hml*^Δ^ >*/*+ on RF (female *n* = 100, 1.4 ± 0.03, male *n* = 100, 1.1 ± 0.08) and *Hml*^Δ^ >*/UAS Pvr*^*Act*^ on RF (female *n* = 100, 1.4 ± 0.06, male *n* = 100, 1.1 ± 0.06). **(l)** Mean intensity of Dilp2quantification. Control, *Hml*^Δ^ >*/*+ on RF (*n* = 9, 446.88 ± 349.05) and *Hml*^Δ^ >*/UAS Pvr*^*Act*^ on RF (*n* = 9, 649.86 ± 678.53). **(m)** Mean intensity of Dilp5 quantification. Control, *Hml*^Δ^ >*/*+ on RF (*n* = 9, 411.82 ± 365.73) and *Hml*^Δ^ >*/UAS Pvr*^*Act*^ on RF (*n* = 8, 474.98 ± 476.43). **(n)** Glucose levels were measured in whole larvae (*Hml*^Δ^ >*/*+, RF, *n* = 18, 0.094 ± 0.036 and *Hml*^Δ^ >*/UAS Pvr*^*Act*^, RF, *n* = 15, 0.08 ± 0.025), hemolymph (*Hml*^Δ^ >*/*+, RF, *n* = 30, 0.09 ± 0.014 and *Hml*^Δ^ >*/UAS Pvr*^*Act*^ RF, *n* = 30, 0.08 ± 0.03), and fat body (*Hml*^Δ^ >*/*+, RF, *n* = 30, 0.08 ± 0.02 and *Hml*^Δ^ >*/UAS Pvr*^*Act*^, RF, *n* = 30, 0.08 ± 0.03). **(o)** Glycogen level measures in whole larvae of *Hml*^Δ^ >*/*+, RF (*n* = 6, 1.08 ± 0.12) and *Hml*^Δ^ >*/UAS Pvr*^*Act*^, RF (*n* = 6, 1.46 ± 0.16, ^***^*p*-value = 0.0009). **(p)** Hemolymph trehalose levels of *Hml*^Δ^ >*/*+, RF (*n* = 66, 0.18 ± 0.05) and *Hml*^Δ^ >*/UAS Pvr*^*Act*^, RF (*n* = 66, 0.17 ± 0.06). **(q–t)** Analysis of fat body insulin signaling in **(q–s)** control (*Hml*^Δ^ >*/*+) on RF and (**q****′****-s****′**) *Hml*^Δ^ >*/UAS Pvr*^*Act*^ on RF shows no change in **(q,q****′****)** tGPH expression, quantified in **(t**, **r,r****′****)** pAKT immunostaining, and **(s,s****′****)** FoxO expression. **(t)** Mean tGPH intensity quantification of control, *Hml*^Δ^ >*/*+ on RF (*n* = 25, 2621 ± 486) and *Hml*^Δ^ >*/UAS Pvr*^*Act*^ on RF (*n* = 25, 2875 ± 158). **(u,u****′****)** Neutral lipid (Nile red) staining in fat bodies of **(u)** *Hml*^Δ^ >*/*+ and **(u****′****)** *Hml*^Δ^ >*/UAS Pvr*^*Act*^ on RF are comparable. **(v)** TAG levels measurements in whole larvae (*Hml*^Δ^ >*/*+ on RF, *n* = 36, 2.01 ± 0.15 and *Hml*^Δ^ >*/UAS Pvr*^*Act*^ on RF, *n* = 36, 1.98 ± 0.3) and hemolymph (*Hml*^Δ^ >*/*+ on RF, *n* = 48, 0.37 ± 0.15 and *Hml*^Δ^ >*/UAS Pvr*^*Act*^ on RF, *n* = 48, 0.23 ± 0.1).

Supplementary Figure 6 In **(a–c)**, scale bar = 1 mm**, (e–f****′****,h–i****′****)** scale bar = 250 μm. In **(d,g,j)**, bar graphs show mean ± standard deviation (SD) and in these panels statistical analysis applied is unpaired *t-*test, two tailed. “*n*” is the total number of larvae analyzed, RF indicates regular food and HSD indicates high-sucrose diet. **(a–d)** Larval images showing blood cells (Hml^+^, GFP, green) in **(a)** control (*Hml*^Δ^ >*/*+), **(b)** *Hml*^Δ^ >*/UAS Pvr*^*WT*^, and **(c)** *Hml*^Δ^ >*/UAS Shi*^*ts*^. Expression of *Pvr*^*WT*^ or *Shi*^*ts*^ in Hml^+^ cells causes increase in GFP-positive cells, quantified in **(d)**. **(d)** Quantifications of total blood cell numbers and Hml^+^ blood cells in *Hml*^Δ^ >*/*+ (total number of blood cells/mm^2^, *n* = 20, 203.49 ± 41.20 and GFP-positive cells, *n* = 20, 152.37 ± 42.6), *Hml*^Δ^ >*/UAS Pvr*^*WT*^ (total number of blood cells/mm^2^, *n* = 10, 403.20 ± 114.54, ^***^*p*-value < 0.0001 and GFP-positive cells, *n* = 10, 334.32 ± 76.49, ^***^*p*-value < 0.0001), and *Hml*^Δ^ >*/UAS Shi*^*ts*^ (total number of blood cells, *n* = 16, 503.40 ± 68.26, ^***^*p*-value < 0.0001 and GFP-positive cells *n* = 16, 430.90 ± 68.14, ^***^*p*-value < 0.0001). **(e–g)** Increasing immune cell numbers of an activated state restores HSD growth defect. Compared with **(e,e**′**)** control wing sizes, *Hml*^Δ^ >*/*+ on HSD **(f,f****′****)**, *Hml*^Δ^ >*UAS Pvr* on HSD showed a significant growth rescue. **(g)** Quantifications of wingspan areas of *Hml*^Δ^ >*/*+ on HSD (female *n* = 50, 1.01 ± 0.07, male *n* = 50, 0.9 ± 0.05) and in *Hml*^Δ^ >*/UAS Pvr*^*WT*^ on HSD (female *n* = 25, 1.09 ± 0.09, ^**^*p*-value = 0.0038, male *n* = 25, 1.03 ± 0.05, ^***^*p*-value < 0.0001). **(h–j)** Increasing immune cell numbers by expressing *UAS Shi*^*ts*^ did not restore HSD growth defect. Compared with **(h,h****′****)** control wing sizes, *Hml*^Δ^ >*/*+ on HSD **(i,i****′****)**, *Hml*^Δ^ >*UAS Shi*^*ts*^ showed a significant growth retardation. **(j)** Quantifications of wing span areas of *Hml*^Δ^ >*/*+ on HSD (female *n* = 50, 1.0 ± 0.1, male *n* = 50, 0.90 ± 0.11) and in *Hml*^Δ^ >*/UAS Shi*^*ts*^ on HSD (female *n* = 50, 0.9 ± 0.09, ^*^*p*-value = 0.0223, male *n* = 50, 0.78 ± 0.09, ^**^*p*-value = 0.0031).

Supplementary Figure 7 In **(c–e,g–i,k–m,o–q)**, scale bar = 250 μm. In **(a,b,f,j,n,r)**, bar graphs show mean ± standard deviation (SD) and statistical analysis applied in these panels is unpaired *t*-test. “*n*” is total number of larvae analyzed, RF is regular food, HSD is high-sucrose diet, HFD is high-fructose diet, and HGD is high-glucose diet. **(a,b)** High-sugar diet causes adult growth retardation. Quantification of wing span areas of **(a)** females (*Hml*^Δ^ >*/*+) and **(b)** males (*Hml*^Δ^ >*/*+) reared on different sugar diets. *Hml*^Δ^ >*/*+ on RF (female *n* = 50, 1.45 ± 0.06 and male *n* = 50, 1.10 ± 0.04), *Hml*^Δ^ >*/*+ on HSD (female *n* = 50, 1.01 ± 0.08, ^***^*p*-value < 0.0001, compared with RF females and male *n* = 50, 0.84 ± 0.13, ^***^*p*-value < 0.0001, compared with RF males), *Hml*^Δ^ >*/*+ on HFD (female *n* = 50, 1.13 ± 0.13, ^***^*p*-value < 0.0001, compared with RF females and male *n* = 50, 0.84 ± 0.07, ^***^*p*-value < 0.0001, compared with RF males), and *Hml*^Δ^ >*/*+ on HGD (female *n* = 25, 0.74 ± 0.08, ^***^*p*-value < 0.0001, compared with RF females and male *n* = 25, 0.60 ± 0.11, ^***^*p*-value < 0.0001, compared with RF males). **(c–r)** Increasing immune cell numbers of an activated state restores growth defect induced by high-sugar diet as represented in wing span sizes. **(c–j)** HFD-induced growth defect seen in **(c,g)** control, *Hml*^Δ^ >*/*+ females and males, is restored in **(d,h)** *Hml*^Δ^ >*/UAS Pvr*^*Act*^ HSD condition but not restored in **(e,i)** *Hml*^Δ^ >*/UAS Shi*^*ts*^ HFD animals. Contrarily, **(e)** *Hml*^Δ^ >*/UAS Shi*^*ts*^ HFD females show a significant growth defect. Quantifications of wing span areas in **(f)** females and **(j)** males. **(f)** Wing span areas of *Hml*^Δ^ >*/*+ HFD (female *n* = 35, 1.13 ± 0.13), *Hml*^Δ^ >*/UAS Pvr*^*Act*^ HFD (female *n* = 51, 1.26 ± 0.08, ^***^*p*-value < 0.0001), and *Hml*^Δ^ >*/UAS Shi*^*ts*^ HFD (female *n* = 25, 0.99 ± 0.09, ^***^*p*-value <0.0002) all comparisons made with *Hml*^Δ^ >*/*+ HFD females. **(j)** Wingspan areas of *Hml*^Δ^ >*/*+ HFD (male *n* = 25, 0.84 ± 0.07), *Hml*^Δ^ >*/UAS Pvr*^*Act*^ HFD (male *n* = 25, 0.97 ± 0.07, ^***^*p*-value < 0.0001), and *Hml*^Δ^ >*/UAS Shi*^*ts*^ HFD (male *n* = 25, 0.83 ± 0.07). All comparisons made with *Hml*^Δ^ >*/*+ HFD males. **(k–r)** HGD-induced growth defect seen in **(k,o)** control, *Hml*^Δ^ >*/*+ females and males, is restored in **(l,p)** *Hml*^Δ^ >*/UAS Pvr*^*Act*^ HGD condition but not restored in **(m,q)** *Hml*^Δ^ >*/UAS Shi*^*ts*^ HGD animals. Quantifications of wing span areas in **(n)** females and **(r)** males. **(n)** Wing span areas of *Hml*^Δ^ >*/*+ HGD (female *n* = 25, 0.74 ± 0.07), *Hml*^Δ^ >*/UAS Pvr*^*Act*^ HGD (female *n* = 25, 0.96 ± 0.09, ^***^*p*-value < 0.0001) and *Hml*^Δ^ >*/UAS Shi*^*ts*^ HGD (female *n* = 25, 0.77 ± 0.06). All comparisons made with *Hml*^Δ^ >*/*+ HGD females. **(r)** Wingspan areas of *Hml*^Δ^ >*/*+ HGD (male *n* = 25, 0.6 ± 0.11), *Hml*^Δ^ >*/UAS Pvr*^*Act*^ HGD (male *n* = 25, 0.9 ± 0.05, ^***^*p*-value < 0.0001), and *Hml*^Δ^ >*/UAS Shi*^*ts*^ HGD (male *n* = 25, 0.61 ± 0.08). All comparisons made with *Hml*^Δ^ >*/*+ HGD males.

Supplementary Figure 8 In **(d**, **e)**, scale bar = 20 μm. In **(a–c,g)**, bar graphs show mean ± standard deviation (SD) and statistical analysis applied in these panels is unpaired *t*-test, two-tailed. “*n*” is the total number of larvae analyzed, a.u is arbitrary unit, FB is fat body, f.c is fold change, and HSD is high-sucrose diet. **(a)** Mean intensity of Dilp2 quantification of representative image shown in Figures 5a,b. Control, *Hml*^Δ^ >*/*+ on HSD (*n* = 5, 2291 ± 678) and *Hml*^Δ^ >*/UAS Pvr*^*Act*^ on HSD (*n* = 5, 1225 ± 578.6, ^*^*p*-value = 0.0283). **(b)** Mean intensity of Dilp5 quantification of representative image shown in Figures 5c,d. Control, *Hml*^Δ^ >*/*+ on HSD (*n* = 5, 1310 ± 304) and *Hml*^Δ^ >*/UAS Pvr*^*Act*^ on HSD (*n* = 5, 580.3 ± 238.7, ^**^*p*-value = 0.0029). **(c)** Fat body glucose levels. *Hml*^Δ^ >*/*+ on HSD (*n* = 35, 0.02 ± 0.017) and *Hml*^Δ^ >*/UAS Pvr*^*Act*^ on HSD (*n* = 35, 0.09 ± 0.03, ^***^*p*-value = 0.0005). **(d–f)** Fat body pAKT analysis on HSD. **(d,e)** Immunostaining of feeding L3 larval fat bodies with anti-pAKT antibody in **(d)** control, *Hml*^Δ^ >*/*+, and **(e)** *Hml*^Δ^ >*/UAS Pvr*^*Act*^ backgrounds shows no change. **(f)** Immunoblot analysis of pAkt/Akt ratio in fat bodies of feeding L3 larvae of control (*Hml*^Δ^ >*/*+) and *Hml*^Δ^ >*/UAS Pvr*^*Act*^ reveals a small increase (fold change ± SD mentioned in the blots). β-Tubulin was used as the internal loading control. **(g)** Fat body analysis of *tobi* m*RNA* levels on HSD. Fold change is represented and statistical analysis was done using **C**_**t**_values (*Hml*^Δ^ >*/*+ on HSD, *n* = 80, 11.83 ± 1.32 and *Hml*^Δ^ >*/UAS Pvr*^*Act*^ on HSD, *n* = 80, 12.11 ± 0.73).
