# Supplementary figures and images for: Immune Control of Animal Growth in Homeostasis and Nutritional Stress in Drosophila
Source: Front Immunol. 2020 Jul 31;11:1528. doi: 10.3389/fimmu.2020.01528 (PMC7416612; doi:10.3389/fimmu.2020.01528)

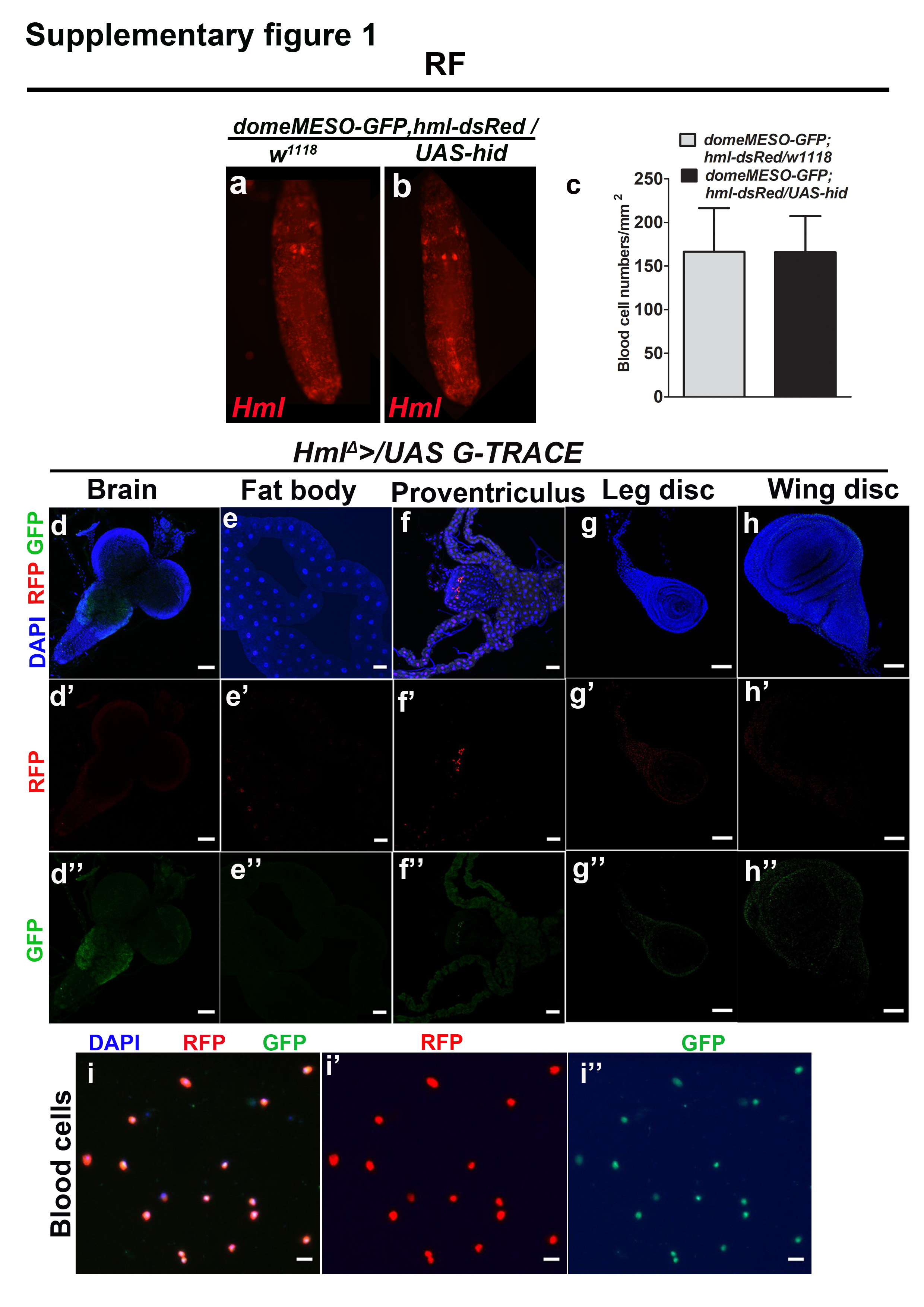

Supplement: Supplementary Figure 1 — In (d–h″), scale bar = 20 μm. Bar graph in (c) shows mean ± standard deviation (SD). Statistical analysis applied in panel c is unpaired t-test, two-tailed in (c). “n” is the total number of larvae analyzed. (a–c) UAS-hid genetic background does not show any blood cell defect. 3rd instar larval images depicting Hml+ blood cells (red, marked with RFP) in (a) control (domeMESO-GFP; hml-dsRed/+) and (b) domeMESO-GFP; hml-dsRed/UAS-hid are comparable. (c) Blood cell counts/mm2 in these backgrounds (domeMESO-GFP; hml-dsRed/w1118, n = 5, 166.5 ± 111.7 and domeMESO-GFP; hml-dsRed/UAS-hid, n = 5, 165.8 ± 92.7). (d–i″) Lineage analysis of HmlΔgal4 using GTRACE confirms blood-specific expression. RFP (real-time HmlΔgal4 expression) or GFP (lineage-based HmlΔgal4 expression) was not detected in (d–d″) brain, (e–e″) fat body, (f–f″) proventriculus, (g–g″) leg disc, (h–h″) wing disc. (i–i″) Both RFP and GFP expression is detected in blood cells. [file Image_1.JPEG]

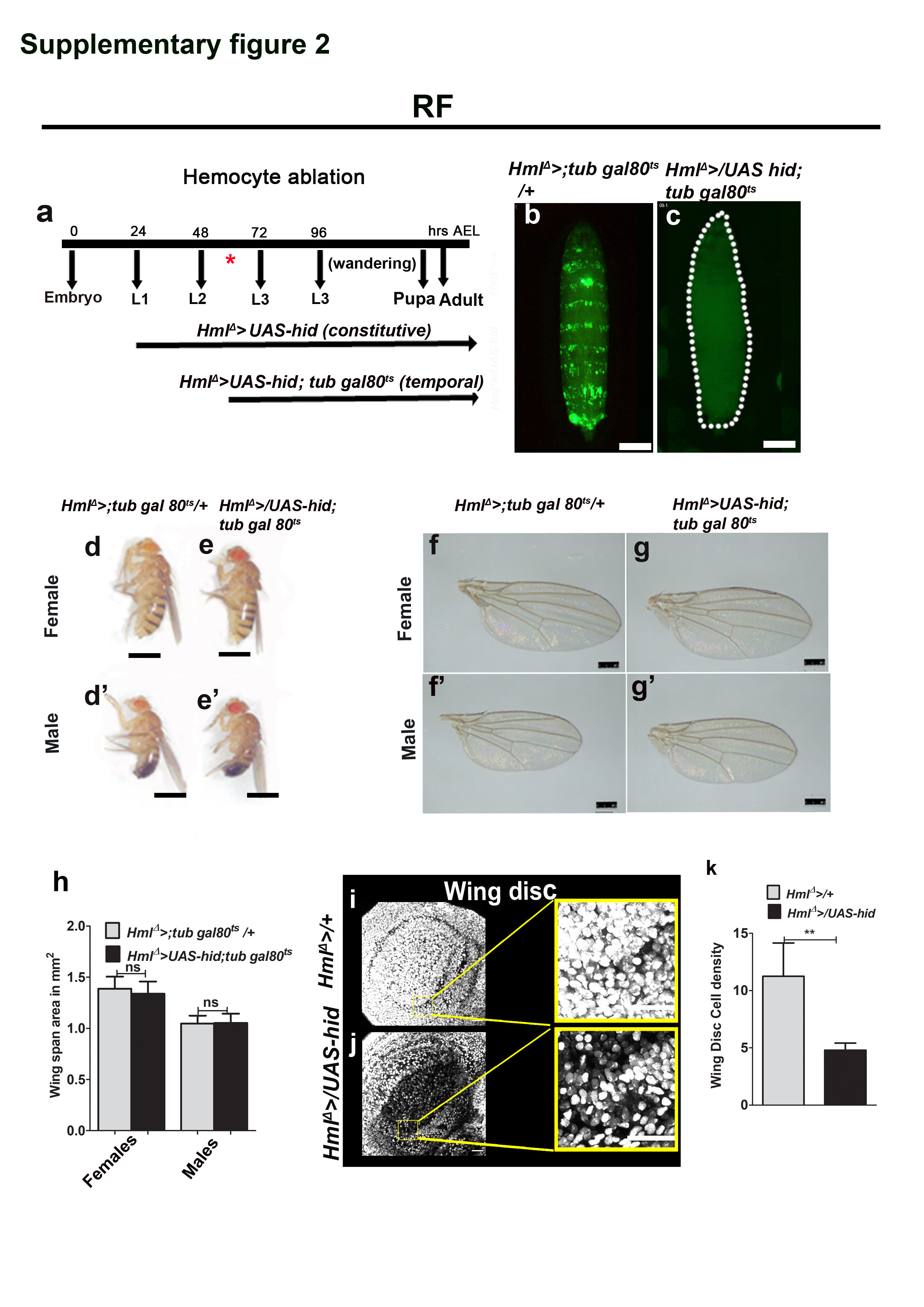

Supplement: Supplementary Figure 2 — In (b,c,d–e′), scale bar = 1 mm, (f–g′) = 250 μm, and (i,j) = 20 μm. In (h, k), bar graphs show mean ± standard deviation (SD). Statistical analysis in (h, k) is unpaired t-test, two-tailed. “n” is the total number of larvae analyzed. (a) Shows the pictorial representation of the constitutive and temporal experiments that have been done using HmlΔ > and HmlΔ >; tub gal80ts, respectively. The red mark indicates the temporal expression of UAS hid in blood cells by shifting the larvae from permissive (18°C) to non-permissive temperature (29°C). (b,c) Temporal expression of UAS-hid in Hml+ blood cells (marked by GFP, green) causes a loss of blood cells. Compared with (b) control (HmlΔ >UAS-GFP; tub gal80ts/+), (c) HmlΔ >UAS-GFP; tub gal80ts/ UAS hid larvae show no GFP signal. (d–h) Temporal expression of UAS-hid in Hml+ blood cells did not alter adult growth. Representative adult (d–e′) fly and (f–g′) wing images in (d, d′, f, f′) control (HmlΔ >UAS-GFP; tub gal80ts/+) and (e,e′,g,g′) HmlΔ >UAS-GFP; tub gal80ts/ UAS hid show no growth reduction. (h) Wingspan area quantifications in HmlΔ >; tub gal80ts/+ (female n = 100, 1.4 ± 0.1, male n = 100, 1.05 ± 0.07) and in HmlΔ >; tub gal80ts/UAS-hid (female n = 100, 1.3 ± 0.1, male n = 100, 1.05 ± 0.09). (i–k) Reduced cell density in wing discs of HmlΔ >/UAS-hid L3 larvae. Compared with cell density seen in wing discs of (i) control (HmlΔ >/+), (j) HmlΔ >/UAS-hid cell density is reduced. (k) Quantified by counting DAPI-positive cells (white). HmlΔ >/+, n = 5, 11.24 ± 3, and HmlΔ >/UAS-hid, n = 5, 4.80 ± 0.60 and (**p-value = 0.0017). [file Image_2.JPEG]

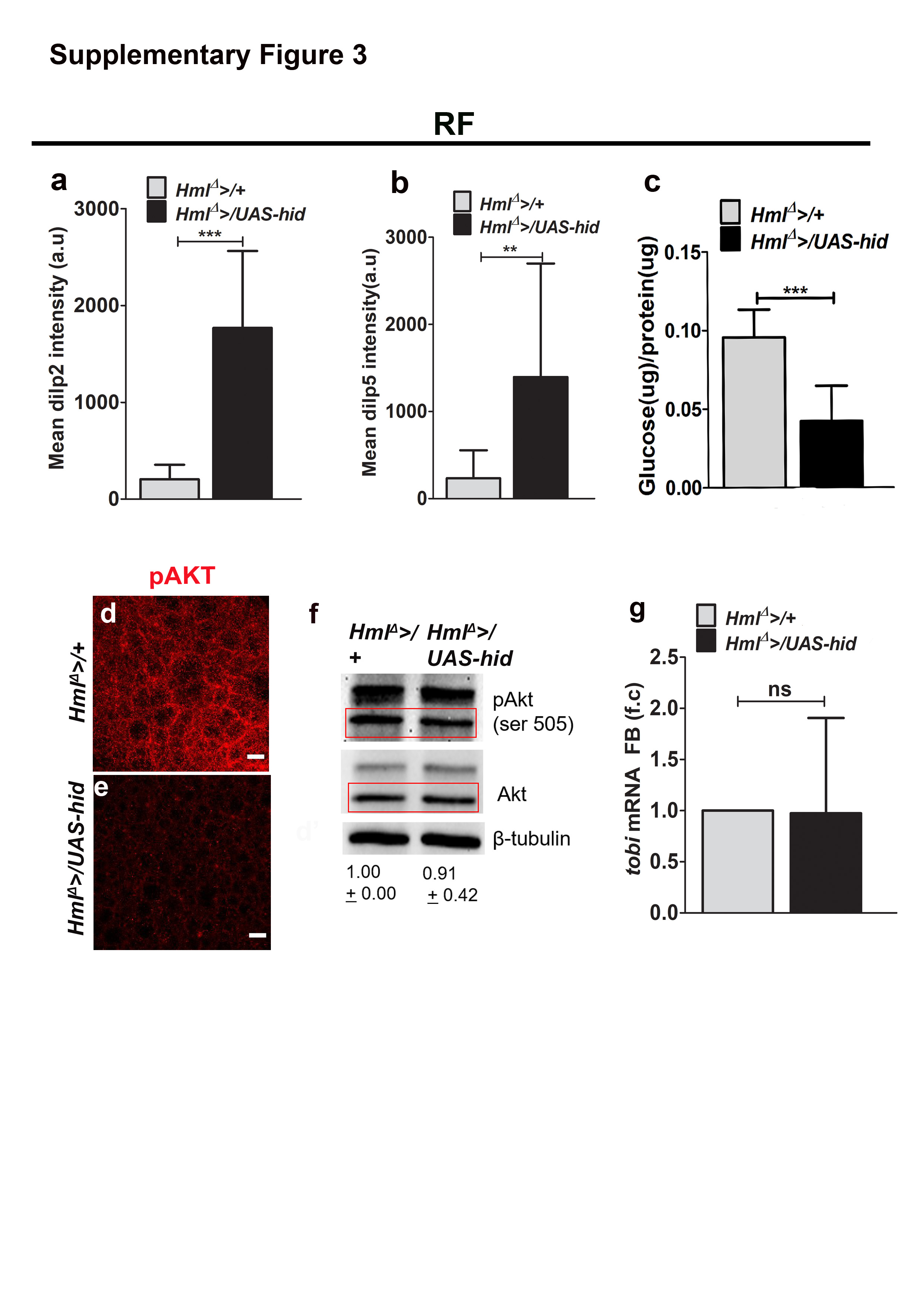

Supplement: Supplementary Figure 3 — In (d,e), scale bar = 20 μm. In (a–c, g), bar graphs show mean ± standard deviation (SD) and statistical analysis applied in these panels is unpaired t-test, two-tailed. “n” is the total number of larvae analyzed, FB is fat body, a.u is arbitrary units, f.c is fold change and RF indicates regular food. (a) Quantification of mean intensity of Dilp2 levels of images shown in Figures 2a,b. Control, HmlΔ >/+ (n = 16, 204.5 ± 149.8) and HmlΔ >/UAS-hid (n = 14, 1768.8 ± 794.2, ***p-value < 0.0001). (b) Quantification of mean intensity of Dilp5 levels of images shown in Figures 2c,d. Control, HmlΔ >/+ (n = 16, 234.023 ± 321.72) and HmlΔ >/UAS-hid (n = 13, 1395.4 ± 1303.9, **p-value = 0.0019). (c) Fat body glucose levels. HmlΔ >/+ (n = 65, 0.095 ± 0.017) and HmlΔ >/UAS-hid (n = 65, 0.04 ± 0.02, ***p-value < 0.0001). (d–f) Fat body pAKT analysis. (d,e) Immunostaining of feeding L3 larval fat bodies with anti-pAKT antibody in (d) control, HmlΔ >/+ and (e) HmlΔ >/UAS-hid backgrounds show reduced pAKT levels in HmlΔ >/UAS-hid condition. (f) Immunoblot analysis of pAkt/Akt ratio in fat bodies of feeding L3 larvae of control (HmlΔ >/+) and HmlΔ >/UAS-hid reveals a small difference (fold change ± SD mentioned in the blots). β-Tubulin was used as the internal loading control. (g) Relative fat body mRNA levels of tobi. Fold change is plotted, and statistical analysis was done using Ctvalues (HmlΔ >/+, n = 60,8.67 ± 3.57 and HmlΔ >/UAS-hid, n = 60, 10.68 ± 0.75). [file Image_3.JPEG]

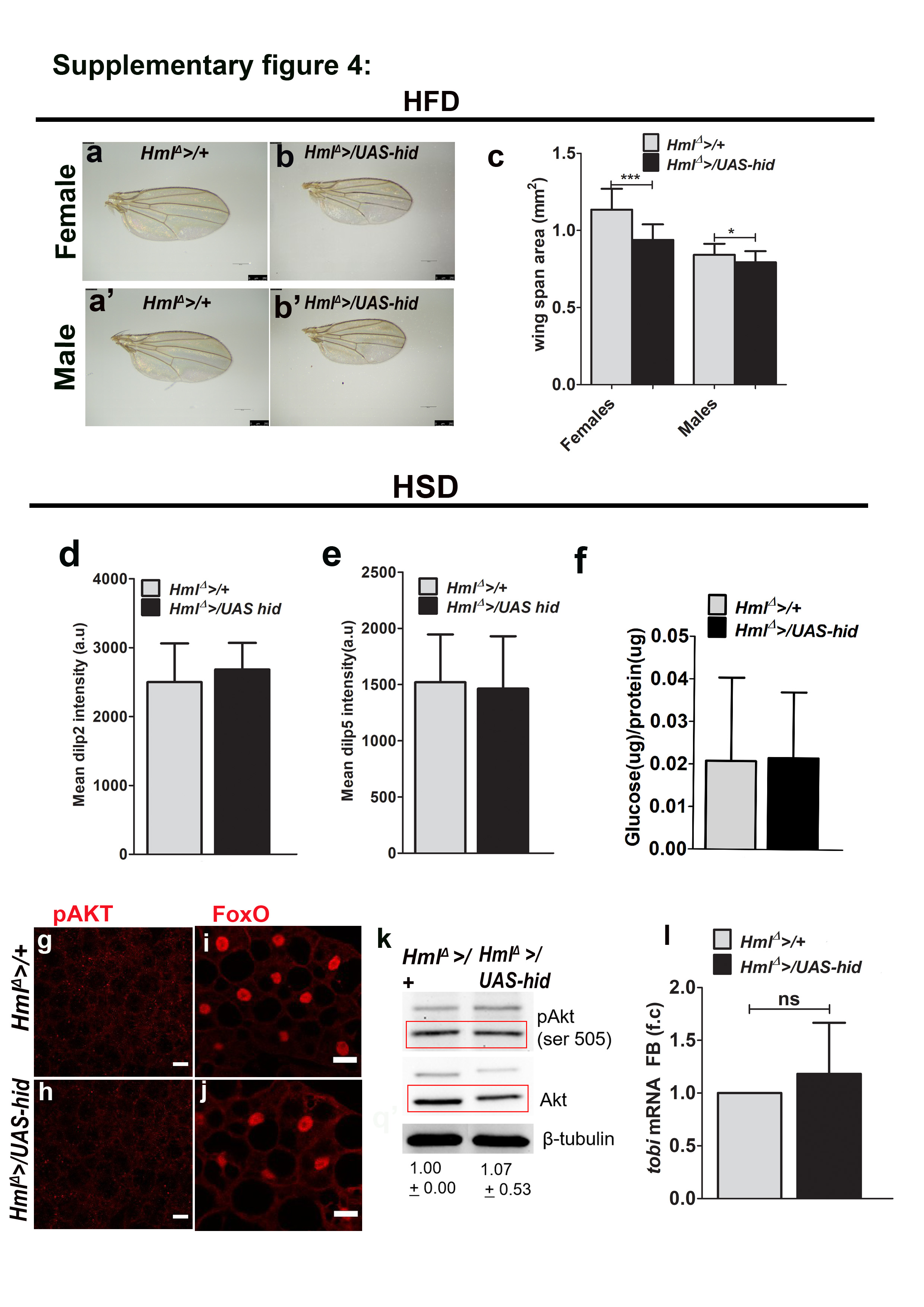

Supplement: Supplementary Figure 4 — In (a–b′), scale bar = 250 μm and (g–j) is 20 μm. In (c–f,l), bar graphs show mean ± standard deviation (SD) and statistical analysis applied for these panels is unpaired t-test, two tailed. “n” is the total number of larvae analyzed, FB is fat body, a.u is arbitrary units, f.c is fold change, HFD is high-fructose diet and HSD is high-sugar diet. (a–c) Loss of immune cells affects tolerance to high fructose diet. (a–b′) Representative wing images of (a,a′) control (HmlΔ >/+) on HFD and (b,b′), HmlΔ >/UAS-hid on HFD showing reduction in wing sizes of HmlΔ >/UAS-hid, HFD animals. (c) Quantification of wingspan areas of HmlΔ >/+ on HFD (female, n = 50, 1.4 ± 0.03, male n = 120, 1.1 ± 0.08) and HmlΔ >/UAS-hid on HFD (female n = 120, 1.4 ± 0.06, ***p-value < 0.0001, compared with HmlΔ >/+ HFD females, male n = 120, 1.1 ± 0.06, *p-value <0.0333, compared with HmlΔ >/+ males). (d) Quantification of mean intensity of Dilp2 expression of representative images shown in Figures 3h,i. Control, HmlΔ >/+on HSD (n = 9, 2499 ± 561) and HmlΔ >/UAS-hid on HSD (n = 8, 2681 ± 387.4). (e) Quantification of mean intensity of Dilp5expression of representative images shown in Figures 3j,k. Control, HmlΔ >/+on HSD (n = 10, 1521 ± 425) and HmlΔ >/UAS-hid on HSD (n = 7, 1463 ± 467). (f) Fat body glucose levels. HmlΔ >/+ on HSD (n = 30, 0.021 ± 0.019) and HmlΔ >/UAS-hid on HSD (n = 30, 0.02 ± 0.01). (g,h) pAKT immunostaining in fat bodies of feeding L3 larvae on HSD of (g) control (HmlΔ >/+) and (h) HmlΔ >/UAS-hid are comparable. (i,j) FoxO immunostaining in fat bodies of feeding L3 larvae on HSD of (i) control (HmlΔ >/+) and (j) HmlΔ >/UAS-hid show similar FoxO nuclear localization. (k) Immunoblot analysis of pAkt/Akt ratio in fat bodies of feeding L3 control (HmlΔ >/+) and HmlΔ >/UAS-hid larvae raised on HSD show no change. Fold change ± SD mentioned in the blots. β-Tubulin was used as the internal loading control. (l) Relative fat body mRNA levels of tobi. Fold change is plotted, and statis [file Image_4.JPEG]

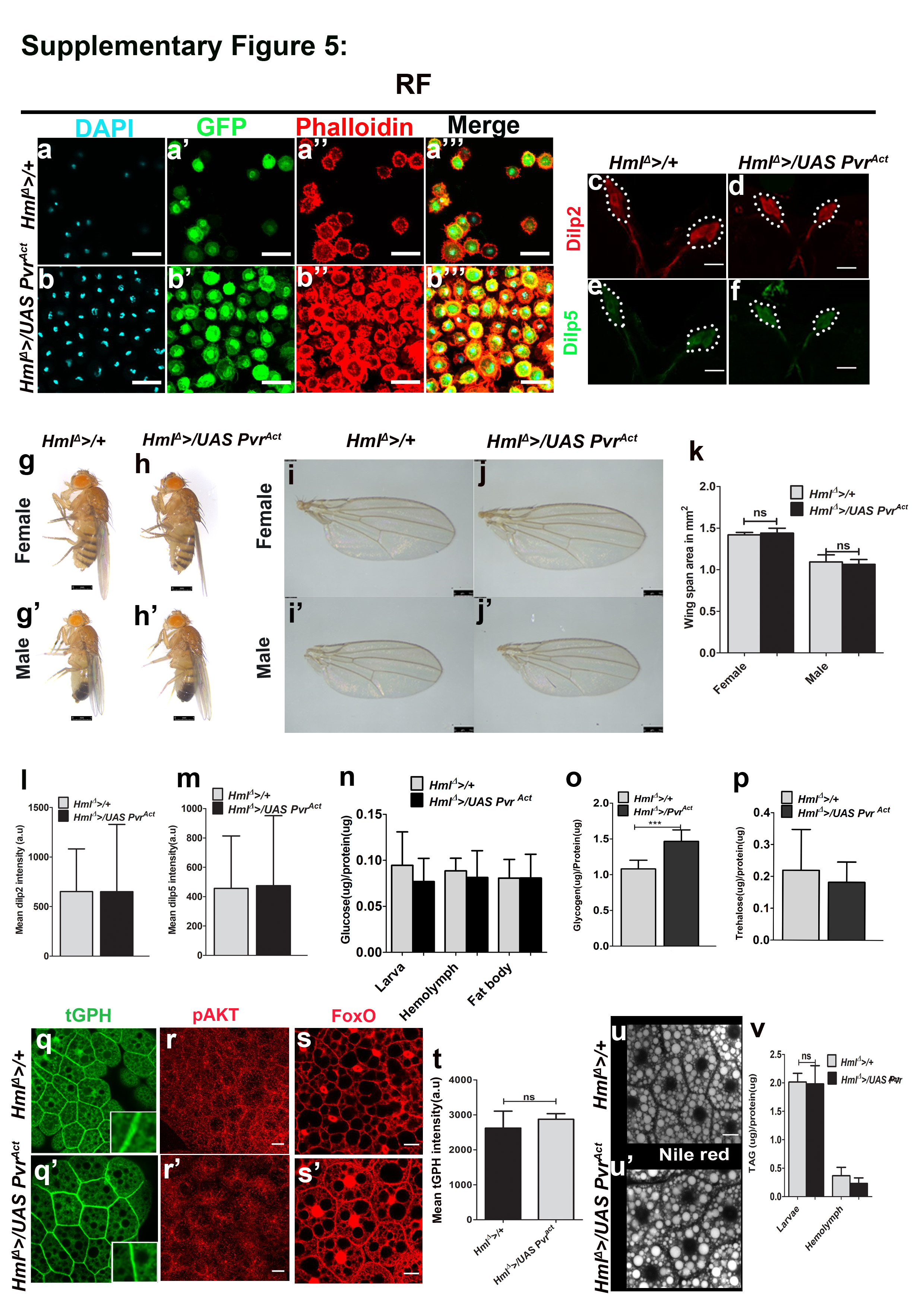

Supplement: Supplementary Figure 5 — In (a–f,l–n′,q–s′), and (u–u′) scale bar = 20 μm, (g–h′) scale bar = 1 mm, and (i–j′) scale bar = 250 μm. In (k,l–p,t,v), bar graphs show mean ± standard deviation (SD) and in these panels statistical analysis applied is unpaired t-test, two tailed. “n” is the total number of larvae analyzed. a.u is arbitrary unit and RF is regular food. (a–b′′′) Characterization of blood cells in HmlΔ >UAS-GFP/UAS PvrAct backgrounds. Compared with blood cells in (a–a′′′) control, HmlΔ >UAS-GFP, (b–b′′′) HmlΔ >UAS-GFP/UAS PvrAct larvae have increased numbers as evident from increased (a,b) DAPI, (a′,b′) Hml (HmlΔ >UAS-GFP) and (a″,b″) phalloidin stainings. (a′′′,b′′′) Merge of all the channels. (c–f) Immunostainings of (c,d) Dilp2 and (e,f) Dilp5 in L3 feeding larval brain insulin-producing cells (IPCs). As compared with (c,e) control (HmlΔ >/+) on RF, (d,f) HmlΔ >/UAS PvrAct on RF, no change in Dilp2 and Dilp5 staining is detected. Quantification of mean intensities in (l,m). (g–k) Expression of UAS PvrAct in Hml+ blood cells have not altered adult growth on RF. Representative adult (g–h′) fly and (i–j′) wing images in (g,g′,i,i′) control (HmlΔ >/+) and (h,h′,j,j′) HmlΔ >/UAS PvrAct show no growth modulation. (k) Quantifications of wing span areas of control HmlΔ >/+ on RF (female n = 100, 1.4 ± 0.03, male n = 100, 1.1 ± 0.08) and HmlΔ >/UAS PvrAct on RF (female n = 100, 1.4 ± 0.06, male n = 100, 1.1 ± 0.06). (l) Mean intensity of Dilp2quantification. Control, HmlΔ >/+ on RF (n = 9, 446.88 ± 349.05) and HmlΔ >/UAS PvrAct on RF (n = 9, 649.86 ± 678.53). (m) Mean intensity of Dilp5 quantification. Control, HmlΔ >/+ on RF (n = 9, 411.82 ± 365.73) and HmlΔ >/UAS PvrAct on RF (n = 8, 474.98 ± 476.43). (n) Glucose levels were measured in whole larvae (HmlΔ >/+, RF, n = 18, 0.094 ± 0.036 and HmlΔ >/UAS PvrAct, RF, n = 15, 0.08 ± 0.025), hemolymph (HmlΔ >/+, RF, n = 30, 0.09 ± 0.014 and HmlΔ >/UAS PvrAct RF, n = 30, 0.08 ± 0.03), and fat body (HmlΔ >/+, RF, n = 30, 0.08 ± 0.02 and HmlΔ >/U [file Image_5.JPEG]

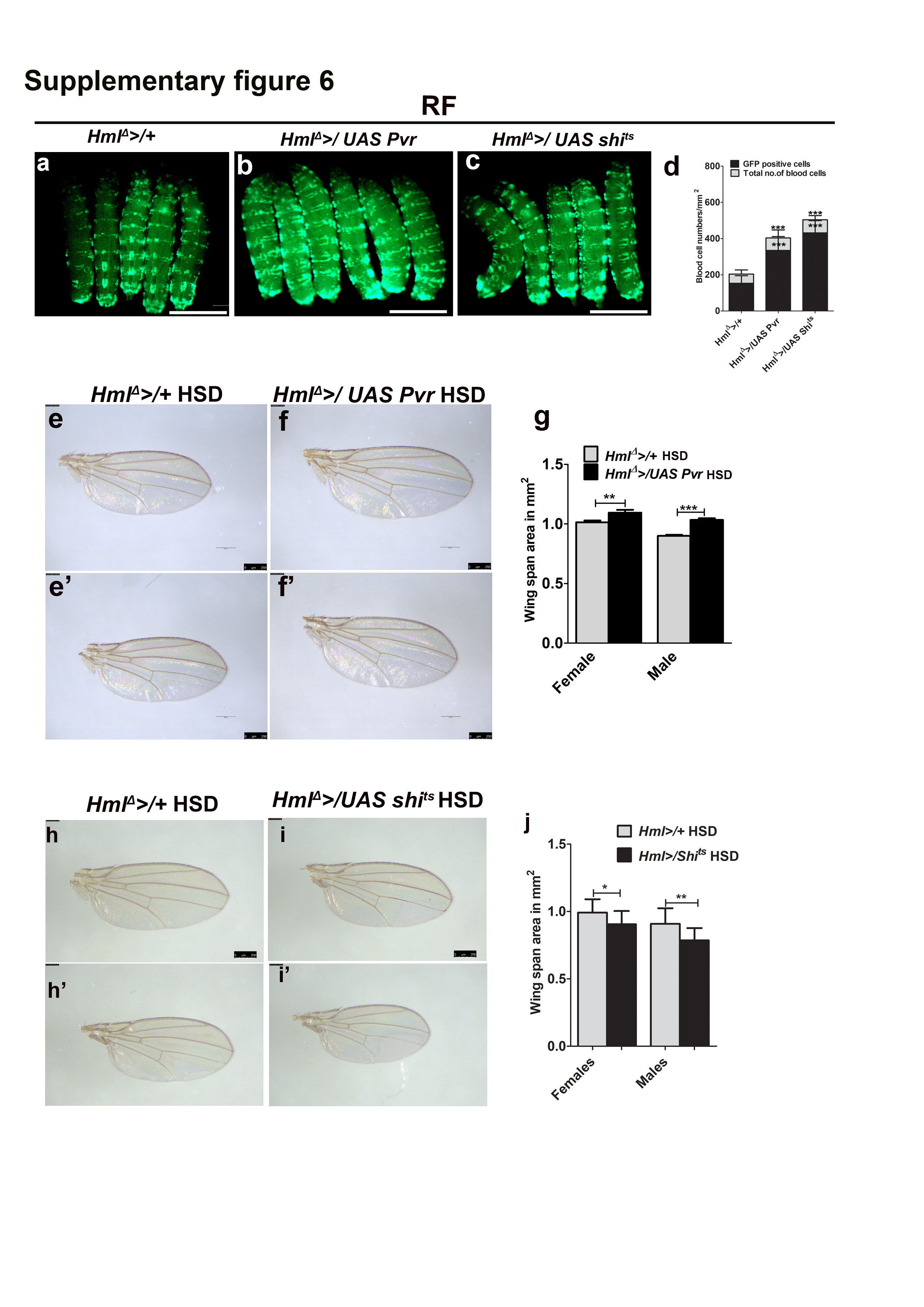

Supplement: Supplementary Figure 6 — In (a–c), scale bar = 1 mm, (e–f′,h–i′) scale bar = 250 μm. In (d,g,j), bar graphs show mean ± standard deviation (SD) and in these panels statistical analysis applied is unpaired t-test, two tailed. “n” is the total number of larvae analyzed, RF indicates regular food and HSD indicates high-sucrose diet. (a–d) Larval images showing blood cells (Hml+, GFP, green) in (a) control (HmlΔ >/+), (b) HmlΔ >/UAS PvrWT, and (c) HmlΔ >/UAS Shits. Expression of PvrWT or Shits in Hml+ cells causes increase in GFP-positive cells, quantified in (d). (d) Quantifications of total blood cell numbers and Hml+ blood cells in HmlΔ >/+ (total number of blood cells/mm2, n = 20, 203.49 ± 41.20 and GFP-positive cells, n = 20, 152.37 ± 42.6), HmlΔ >/UAS PvrWT (total number of blood cells/mm2, n = 10, 403.20 ± 114.54, ***p-value < 0.0001 and GFP-positive cells, n = 10, 334.32 ± 76.49, ***p-value < 0.0001), and HmlΔ >/UAS Shits (total number of blood cells, n = 16, 503.40 ± 68.26, ***p-value < 0.0001 and GFP-positive cells n = 16, 430.90 ± 68.14, ***p-value < 0.0001). (e–g) Increasing immune cell numbers of an activated state restores HSD growth defect. Compared with (e,e′) control wing sizes, HmlΔ >/+ on HSD (f,f′), HmlΔ >UAS Pvr on HSD showed a significant growth rescue. (g) Quantifications of wingspan areas of HmlΔ >/+ on HSD (female n = 50, 1.01 ± 0.07, male n = 50, 0.9 ± 0.05) and in HmlΔ >/UAS PvrWT on HSD (female n = 25, 1.09 ± 0.09, **p-value = 0.0038, male n = 25, 1.03 ± 0.05, ***p-value < 0.0001). (h–j) Increasing immune cell numbers by expressing UAS Shits did not restore HSD growth defect. Compared with (h,h′) control wing sizes, HmlΔ >/+ on HSD (i,i′), HmlΔ >UAS Shits showed a significant growth retardation. (j) Quantifications of wing span areas of HmlΔ >/+ on HSD (female n = 50, 1.0 ± 0.1, male n = 50, 0.90 ± 0.11) and in HmlΔ >/UAS Shits on HSD (female n = 50, 0.9 ± 0.09, *p-value = 0.0223, male n = 50, 0.78 ± 0.09, **p-value = 0.0031). [file Image_6.JPEG]

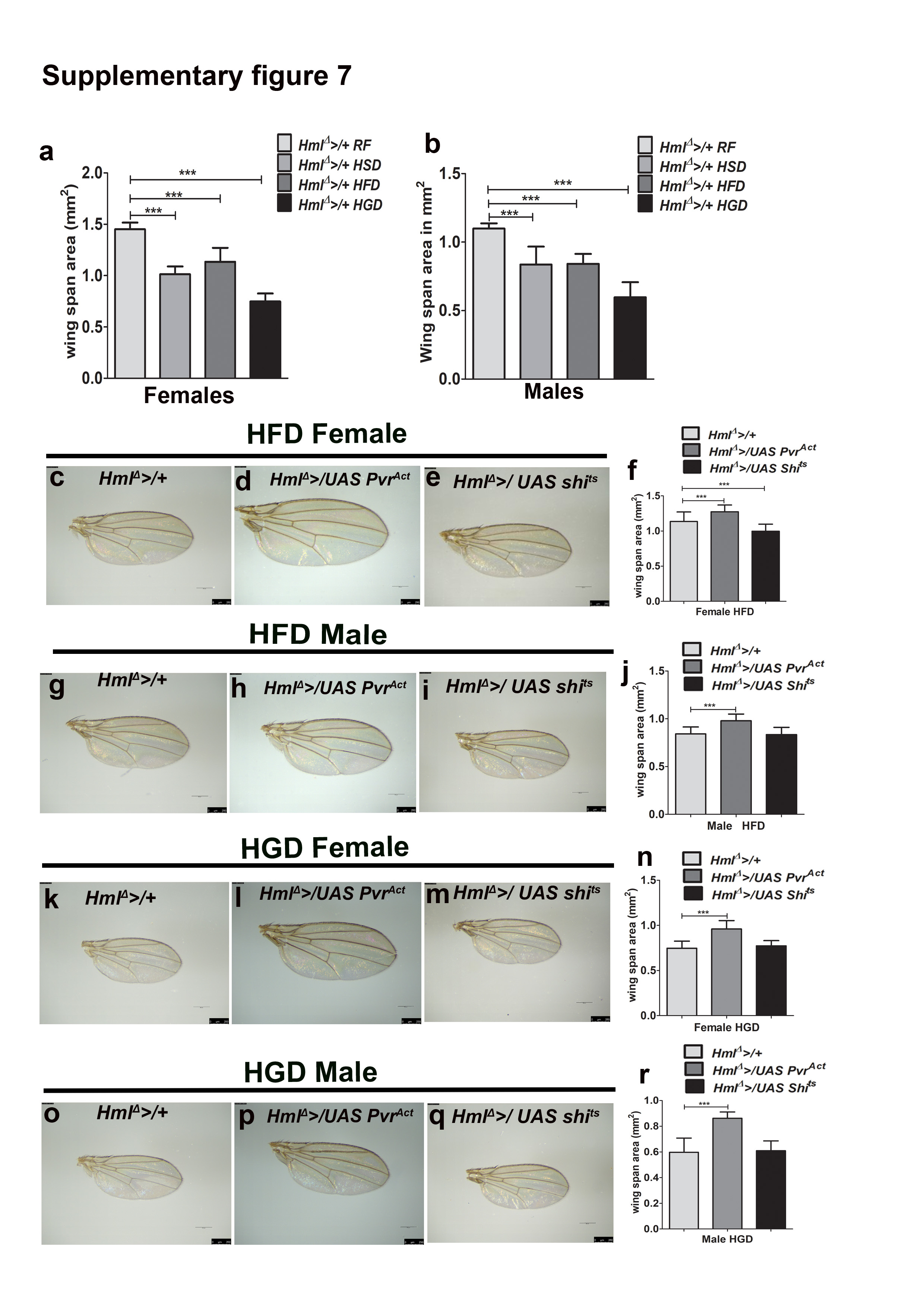

Supplement: Supplementary Figure 7 — In (c–e,g–i,k–m,o–q), scale bar = 250 μm. In (a,b,f,j,n,r), bar graphs show mean ± standard deviation (SD) and statistical analysis applied in these panels is unpaired t-test. “n” is total number of larvae analyzed, RF is regular food, HSD is high-sucrose diet, HFD is high-fructose diet, and HGD is high-glucose diet. (a,b) High-sugar diet causes adult growth retardation. Quantification of wing span areas of (a) females (HmlΔ >/+) and (b) males (HmlΔ >/+) reared on different sugar diets. HmlΔ >/+ on RF (female n = 50, 1.45 ± 0.06 and male n = 50, 1.10 ± 0.04), HmlΔ >/+ on HSD (female n = 50, 1.01 ± 0.08, ***p-value < 0.0001, compared with RF females and male n = 50, 0.84 ± 0.13, ***p-value < 0.0001, compared with RF males), HmlΔ >/+ on HFD (female n = 50, 1.13 ± 0.13, ***p-value < 0.0001, compared with RF females and male n = 50, 0.84 ± 0.07, ***p-value < 0.0001, compared with RF males), and HmlΔ >/+ on HGD (female n = 25, 0.74 ± 0.08, ***p-value < 0.0001, compared with RF females and male n = 25, 0.60 ± 0.11, ***p-value < 0.0001, compared with RF males). (c–r) Increasing immune cell numbers of an activated state restores growth defect induced by high-sugar diet as represented in wing span sizes. (c–j) HFD-induced growth defect seen in (c,g) control, HmlΔ >/+ females and males, is restored in (d,h) HmlΔ >/UAS PvrAct HSD condition but not restored in (e,i) HmlΔ >/UAS Shits HFD animals. Contrarily, (e) HmlΔ >/UAS Shits HFD females show a significant growth defect. Quantifications of wing span areas in (f) females and (j) males. (f) Wing span areas of HmlΔ >/+ HFD (female n = 35, 1.13 ± 0.13), HmlΔ >/UAS PvrAct HFD (female n = 51, 1.26 ± 0.08, ***p-value < 0.0001), and HmlΔ >/UAS Shits HFD (female n = 25, 0.99 ± 0.09, ***p-value <0.0002) all comparisons made with HmlΔ >/+ HFD females. (j) Wingspan areas of HmlΔ >/+ HFD (male n = 25, 0.84 ± 0.07), HmlΔ >/UAS PvrAct HFD (male n = 25, 0.97 ± 0.07, ***p-value < 0.0001), and HmlΔ >/UAS Shits HFD (male n = 25, 0.83 ± 0.07). A [file Image_7.JPEG]

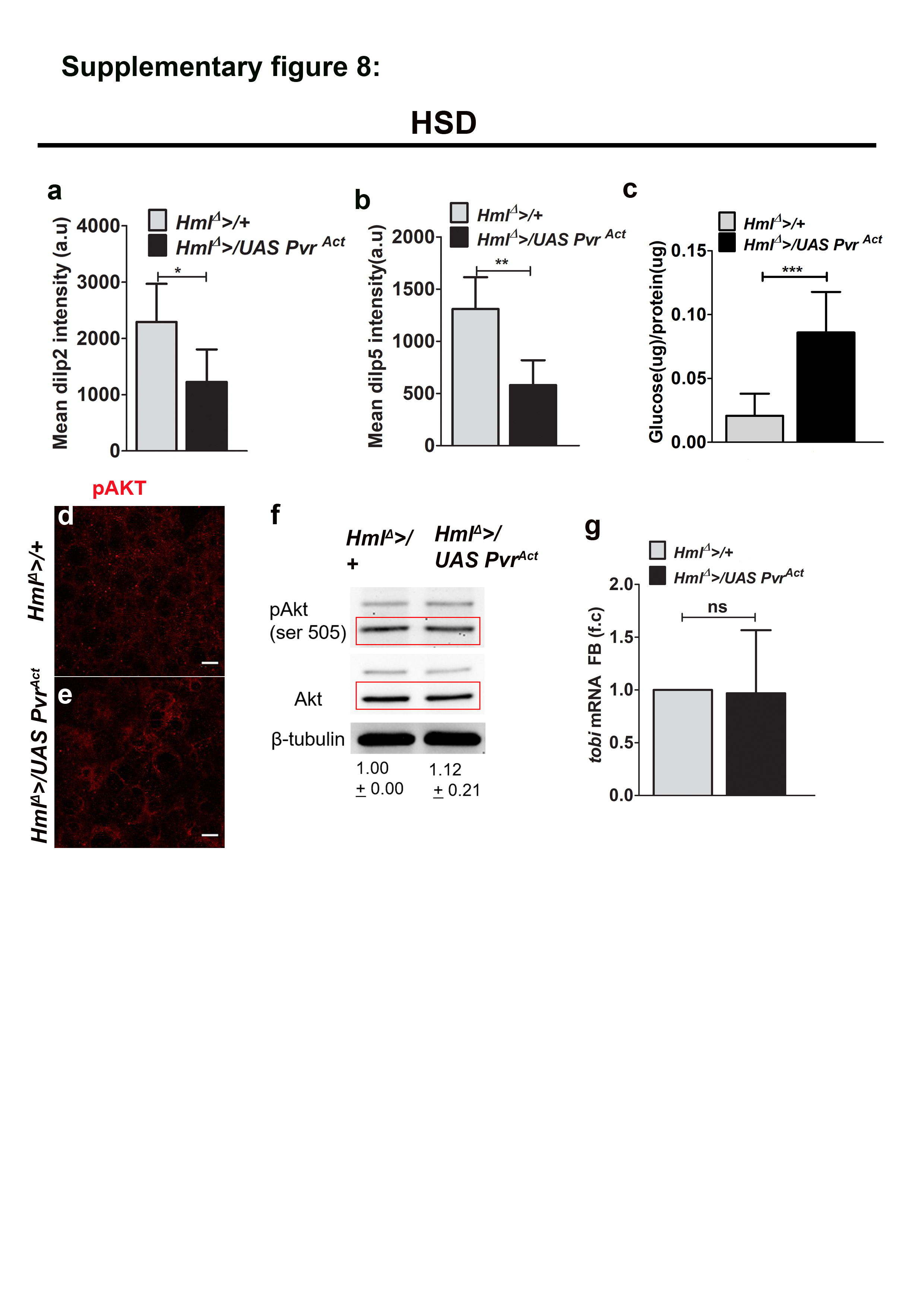

Supplement: Supplementary Figure 8 — In (d, e), scale bar = 20 μm. In (a–c,g), bar graphs show mean ± standard deviation (SD) and statistical analysis applied in these panels is unpaired t-test, two-tailed. “n” is the total number of larvae analyzed, a.u is arbitrary unit, FB is fat body, f.c is fold change, and HSD is high-sucrose diet. (a) Mean intensity of Dilp2 quantification of representative image shown in Figures 5a,b. Control, HmlΔ >/+ on HSD (n = 5, 2291 ± 678) and HmlΔ >/UAS PvrAct on HSD (n = 5, 1225 ± 578.6, *p-value = 0.0283). (b) Mean intensity of Dilp5 quantification of representative image shown in Figures 5c,d. Control, HmlΔ >/+ on HSD (n = 5, 1310 ± 304) and HmlΔ >/UAS PvrAct on HSD (n = 5, 580.3 ± 238.7, **p-value = 0.0029). (c) Fat body glucose levels. HmlΔ >/+ on HSD (n = 35, 0.02 ± 0.017) and HmlΔ >/UAS PvrAct on HSD (n = 35, 0.09 ± 0.03, ***p-value = 0.0005). (d–f) Fat body pAKT analysis on HSD. (d,e) Immunostaining of feeding L3 larval fat bodies with anti-pAKT antibody in (d) control, HmlΔ >/+, and (e) HmlΔ >/UAS PvrAct backgrounds shows no change. (f) Immunoblot analysis of pAkt/Akt ratio in fat bodies of feeding L3 larvae of control (HmlΔ >/+) and HmlΔ >/UAS PvrAct reveals a small increase (fold change ± SD mentioned in the blots). β-Tubulin was used as the internal loading control. (g) Fat body analysis of tobi mRNA levels on HSD. Fold change is represented and statistical analysis was done using Ctvalues (HmlΔ >/+ on HSD, n = 80, 11.83 ± 1.32 and HmlΔ >/UAS PvrAct on HSD, n = 80, 12.11 ± 0.73). [file Image_8.JPEG]
